# Treatment of Denervated Muscle Atrophy by Injectable Dual‐Responsive Hydrogels Loaded with Extracellular Vesicles

**DOI:** 10.1002/advs.202412248

**Published:** 2025-01-21

**Authors:** Ziheng Bu, Jianxing Jing, Wei Liu, Zhen Fan, Junchao Huang, Zheng Zhou, Jianhai Hu, Jinxi An, Jiachang Hong, Jianing Yu, Daolin Tang, Min Sun, Jianzhong Du, Peng Wu

**Affiliations:** ^1^ Department of Orthopedics Shanghai Tenth People's Hospital School of Medicine Tongji University Shanghai 200072 China; ^2^ Department of Polymeric Materials School of Materials Science and Engineering Tongji University 4800 Caoan Road Shanghai 201804 China; ^3^ School of Medicine Anhui University of Science & Technology 168 Taifeng Street, Shannan New District Huainan Anhui 232000 China; ^4^ Department of Surgery UT Southwestern Medical Center Dallas Texas 75390 USA; ^5^ Key Laboratory for Ultrafine Materials of Ministry of Education School of Materials Science and Engineering East China University of Science and Technology Shanghai 200237 China

**Keywords:** denervated muscle atrophy, extracellular vesicles, hydrogel, ultrasound responsiveness

## Abstract

Denervated muscle atrophy, a common outcome of nerve injury, often results in irreversible fibrosis due to the limited effectiveness of current therapeutic interventions. While extracellular vesicles (EVs) offer promise for treating muscle atrophy, their therapeutic potential is hindered by challenges in delivery and bioactivity within the complex microenvironment of the injury site. To address this issue, an injectable hydrogel is developed that is responsive to both ultrasound and pH, with inherent anti‐inflammatory and antioxidant properties, designed to improve the targeted delivery of stem cell‐derived EVs. This hydrogel system allows for controlled release of EVs from human umbilical cord mesenchymal stem cells (HUC‐MSCs), adapting to the specific conditions of the injury environment. In vivo studies using a rat model of nerve injury demonstrated that the EV‐loaded hydrogel (EVs@UR‐gel) significantly preserved muscle function. Six weeks post‐nerve reconstruction, treated rats exhibited muscle strength, circumference, and wet weight reaching 89.53 ± 0.96%, 76.02 ± 7.49%, and 88.0 ± 2.65% of healthy controls, respectively, alongside an improvement in the sciatic nerve index (−0.11 ± 0.09). This platform presents a novel therapeutic approach by maintaining EV bioactivity, enabling tunable release based on the disease state, and facilitating the restoration of muscle structure and function.

## Graphical abstract

This study develops an injectable ultrasound‐responsive hydrogel containing human umbilical cord mesenchymal stem cell extracellular vesicles (EVs). It clears reactive oxygen species (ROS) in target muscles post‐nerve injury. Later, controlled EV release promotes skeletal muscle regeneration and extracellular matrix remodeling, maintaining muscle structure and function.

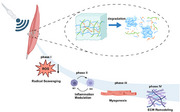

## 1 Introduction

Skeletal muscles are essential for the human motion, facilitating postural control and generating movement.^[^ ^1^, ^2^ ^]^ Muscle atrophy due to nerve damage is prevalent and linked to disrupted nerve supply, and it contributes significantly to global disability and healthcare costs.^[^ ^3^ ^]^ While early nerve repair can mitigate damage, challenges such as suboptimal surgical conditions and slow nerve regeneration often lead to muscle atrophy.^[^ ^4^, ^5^ ^]^ Current treatment approaches focus primarily on neural repair, neglecting the atrophied target muscle.^[^ ^6^, ^7^, ^8^, ^9^ ^]^ Conventional methods, such as passive skeletal muscle exercises and electrical stimulation, fail to meet the clinical needs for preventing atrophy progression.^[^ ^10^, ^11^, ^12^ ^]^

Denervated muscle undergoes a staged, highly programmed pathological process, which, if unchecked, results in irreversible atrophy and lifelong motor dysfunction.^[^ ^13^, ^14^, ^15^, ^16^, ^17^, ^18^, ^19^ ^]^ In this program, the acidic microenvironment caused by early hypoxia and the lack of skeletal muscle regeneration in late stages have the most significant impact on the repair of target muscles. Current therapies often target isolated stages of the disease, lacking a comprehensive strategy to address muscle atrophy from multiple angles. Mesenchymal stem cell (MSC) therapy has shown potential in treating muscle atrophy, but issues such as cell source, tissue origin, and cellular heterogeneity remain obstacles.^[^ ^20^, ^21^, ^22^, ^23^, ^24^, ^25^ ^]^ In contrast, extracellular vesicles (EVs) derived from stem cells offer comparable therapeutic benefits without the complexities associated with live cells.^[^ ^26^, ^27^ ^]^ EVs contain microRNAs (miRNAs), non‐coding RNAs (ncRNAs), and growth factors, which play an important role in cell communication and regulation of cell function.^[^ ^28^, ^29^, ^30^ ^]^ These components are enriched in pathways related to cellular metabolism and may reverse atrophic changes, with their reparative effects showing dose dependency.^[^ ^31^, ^32^, ^33^, ^34^, ^35^, ^36^ ^]^ Our previous study also indicates that higher EV concentrations improve treatment outcomes for bone and cartilage injuries compared to lower doses.^[^ ^29^, ^37^ ^]^

However, the in vivo stability and bioavailability of EVs remain challenges, necessitating their encapsulation in hydrogels to enable tunable release and sustained bioactivity.^[^ ^38^, ^39^, ^40^, ^41^, ^42^ ^]^ In the clinical application of EVs for the treatment of denervated muscle atrophy, it is necessary to address the protection of EVs from being internalized by inflammatory cells, and the ability to controlled release EVs at specific stages. Bioengineering approaches for musculoskeletal injuries show promise, but muscle repair requires materials with high compliance and fatigue resistance, properties still under investigation.^[^ ^43^, ^44^, ^45^ ^]^ Injectable hydrogels have demonstrated potential to respond to mechanical stress via mechanochemical conversion, allowing sol‐to‐gel transitions that facilitate tissue filling, curing, and controlled drug release.^[^ ^46^, ^47^ ^]^ In the context of denervated muscle atrophy, the condition's distinct pathophysiology necessitates biomaterials that are injectable, anti‐inflammatory, antioxidative, and capable of dynamically regulating EV release in response to therapeutic progress.

In this study, we addressed these clinical challenges by demonstrating that extracellular vesicles derived from human umbilical cord mesenchymal stem cells (HUC‐MSC‐EVs) promote myoblast migration, proliferation, and differentiation. And our hydrogel is designed with dual responsiveness to pH and ultrasound, leveraging the Schiff base reactions for pH sensitivity and the mechanical effects of ultrasound on disulfide bonds for controlled EVs release, enhancing treatment efficacy in combating muscle atrophy while preserving muscle function and morphology. This approach combines bioactive materials with therapeutic EVs to target the pathophysiological processes underlying denervated muscle atrophy.

## 2 Results

### 2.1 Synthesis and Characterizations of UR‐Gel

**Scheme 1:**
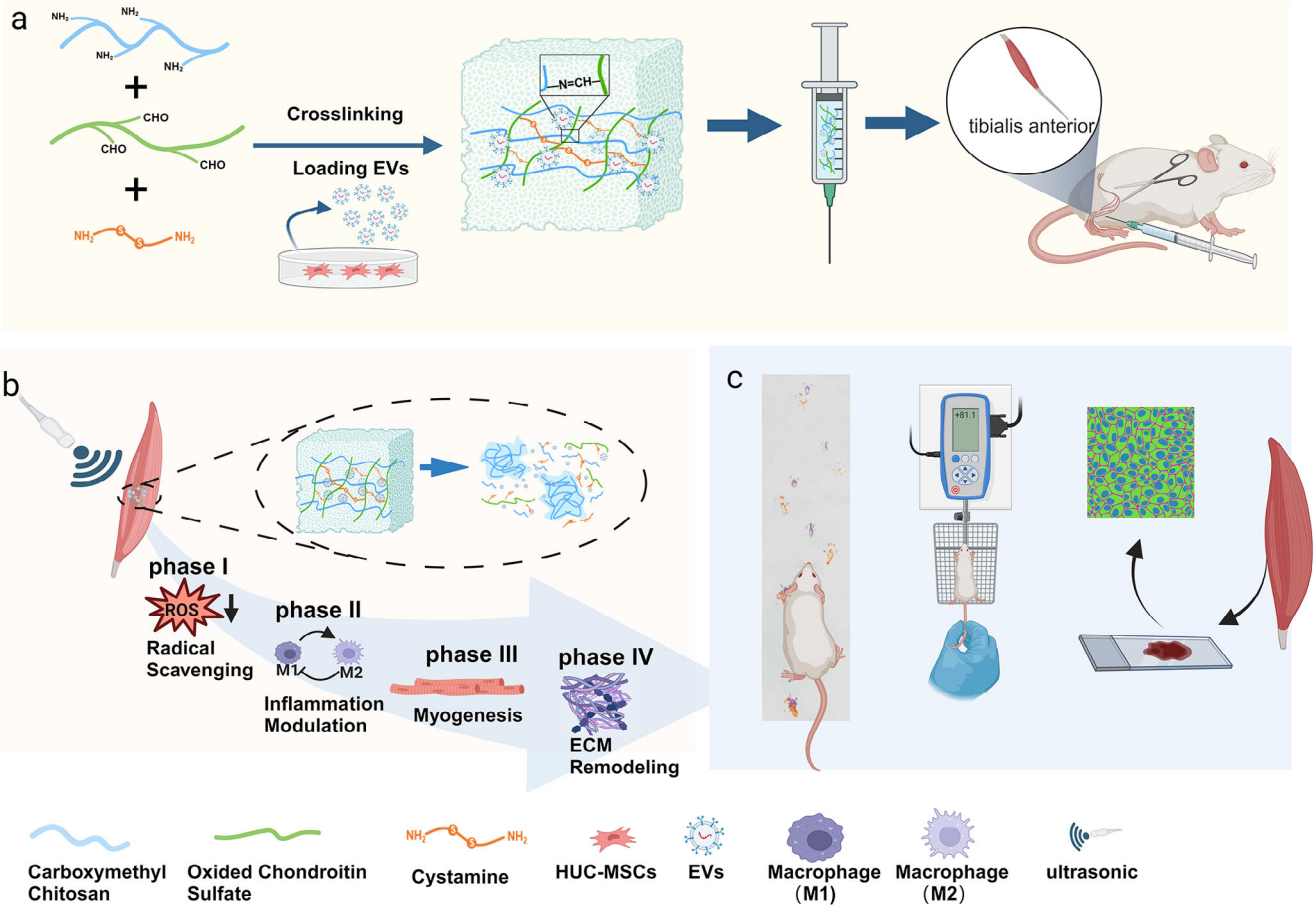
The illustration of bioactive EVs loading dual‐responsive hydrogel used in the staged treatment of denervated muscle atrophy. a) The preparation process of pH/ultrasound dual‐responsive and HUC‐MSCs‐EVs loaded injectable hydrogel and its in vivo administration method. b) The programmed treatment of HUC‐MSCs‐EVs in denervated muscle atrophy model with sonication. In the initial phase of denervated muscle atrophy, the hydrogel scavenged ROS generated by hypoxia. Subsequently, during the second phase of inflammatory response, the hydrogel facilitates inflammatory remodeling. In the third and fourth phases, subsequent to sonication, a considerable number of EVs were released, which facilitated the remodeling of the adult muscle and extracellular matrix to maintain muscle function and prevent irreversible muscle atrophy. c) The therapeutic efficacy of the material was analyzed in terms of the functional and anatomical structure of the skeletal muscle by rat footprint analysis, maximum muscle tone measurements, and immunofluorescence staining of the specimens. (Created with bioRender.com).

**Figure 1:**
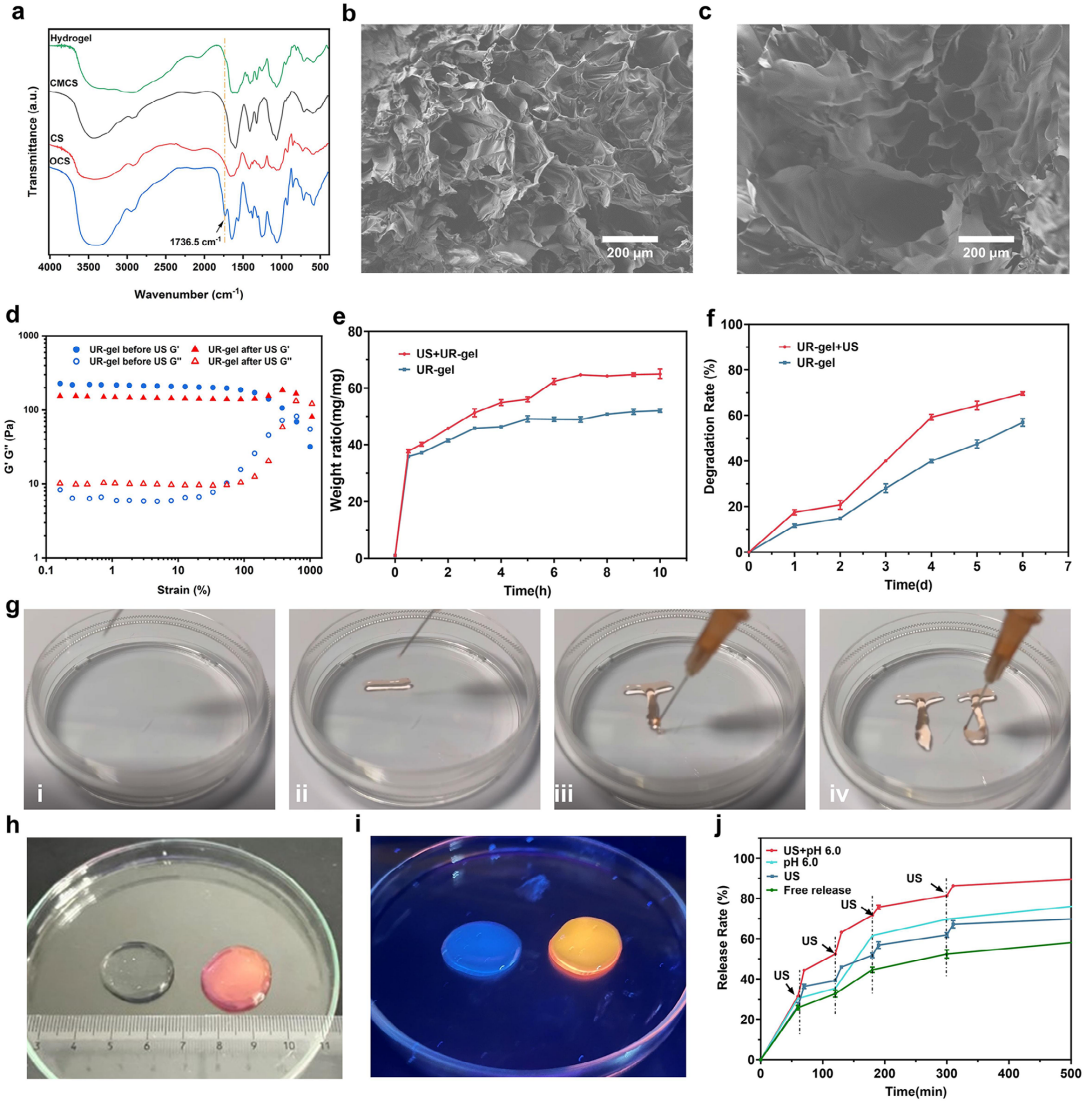
Characterizations and ultrasound‐responsiveness study of UR‐gel. a) FTIR spectra of OCS, CS, CMCS, and UR‐gel. b‐c) The SEM images of UR‐gel (b) before ultrasound stimulation and (c) after ultrasound stimulation. d) Rheological property of hydrogels with different treatments. e) Swelling ratio of ultrasonic treated UR‐gel and UR‐gel immersed in PBS buffer (pH 7.4). f) Degradation rate of ultrasonic treated UR‐gel and UR‐gel in PBS buffer containing lysozyme. g) The demonstration of injectable properties. h) Optical images of the UR‐gel and Dox‐loaded UR‐gel. i) Optical images of the UR‐gel and DOX‐loaded UR‐gel under ultraviolet light. j) The drug release behavior of UR‐gel under different conditions, and the accelerated release was observed at each ultrasound‐stimulation time point.

The UR‐gel is a stimuli‐responsive hydrogel designed to react to pH changes and ultrasound exposure. These hydrogels are engineered to alter their physical or chemical properties, such as swelling, degradation, or drug release, in response to external stimuli, making them valuable for targeted drug delivery, tissue engineering, and regenerative medicine.^[^ ^48^, ^49^ ^]^ The synthesis of UR‐gel outlined in **Scheme** **1**. Oxidized chondroitin sulfate (OCS) was first produced from chondroitin sulfate (CS) via NaIO_4_ oxidation. Successful oxidation was confirmed by the appearance of the aldehyde stretching vibration band at 1736.5 cm^−1^ in the FTIR spectrum of OCS (**Figure** **1a**). UR‐gel was then synthesized by mixing OCS, carboxymethyl chitosan (CMCS), and cystamine dihydrochloride solutions, where in situ crosslinking occurred via Schiff base reactions between amino and aldehyde groups. The disappearance of the 1736.5 cm^−1^ aldehyde peak in the FTIR spectrum of UR‐gel confirmed complete crosslinking and successful formation of the hydrogel.

SEM imaging (Figure 1b) revealed a porous network structure in the hydrogel, suitable for encapsulating EVs. Upon ultrasound stimulation, pore size increased (Figure 1c), likely due to partial disruption of the crosslinked structure. Rheological analysis further supported these findings, as the storage modulus (G′) exceeded the loss modulus (*G″*) at low strain (0.1%). At higher strain (1002.14%), *G′* was lower than *G″*, indicating structural degradation. Ultrasound stimulation reduced *G′*, confirming that ultrasound disrupts the hydrogel structure and accelerates degradation (Figure 1d).

The swelling study demonstrated the hydrogel's capacity to absorb large amounts of water, maintaining a water absorption rate of ≈50 times its original volume after 8 h (Figure 1e). This indicates excellent swelling performance and drug storage capacity. Biodegradability was assessed in PBS buffer with lysozyme, showing a degradation rate of 69.92% after 6 days of immersion (Figure 1f). The reversible covalent crosslinking also enabled injection capability (Figure 1g).

The drug release behavior of UR‐gel was evaluated using doxorubicin (DOX) as a model drug (Figure 1h). Fluorescence imaging under ultraviolet light (465 nm) confirmed successful drug loading into the hydrogel (Figure 1i). The release kinetics were further investigated using doxorubicin as a model drug. The release rate was determined using a standardized calibration curve of doxorubicin at various concentrations (Figure S1a–b, Supporting Information). As shown in Figure 1j, drug release from UR‐gel was significantly enhanced under ultrasound stimulation or acidic conditions (pH 6.0) compared to free diffusion. Moreover, the combination of ultrasound stimulation and acidic environment synergistically accelerated drug release, reaching 92.35% after 420 min of incubation.

Infrared thermography is used to detect the thermal effect of ultrasound on materials (Figure S1c–d, Supporting Information). By comparing the temperature changes of simulated body fluid (SBF) and UR‐gel, the thermal effect of ultrasound on UR‐gel can be inferred. The results showed that there was no difference in temperature changes between SBF and UR gel after 10 min of intervention under 1 MHz ultrasound, with the temperature reaching equilibrium from room temperature of 25 °C to 39 °C.

Collectively, these results demonstrate the successful synthesis, structural integrity, and functional performance of UR‐gel.

### 2.2 Isolation and Characterization of EVs Derived from HUC‐MSCs

HUC‐MSCs are a type of mesenchymal stem cell isolated from Wharton's jelly in the human umbilical cord. These cells possess multipotent differentiation capacity, enabling them to differentiate into various cell types, including chondrocytes (cartilage), osteocytes (bone), and adipocytes (fat). Light microscopy revealed the typical spindle‐shaped morphology characteristic of HUC‐MSCs (Figure S2a, Supporting Information). The differentiation potential of HUC‐MSCs was further validated by Alcian blue staining for cartilage, Alizarin Red staining for bone, and Oil Red O staining for adipose tissue (Figure S2b, Supporting Information). Flow cytometry analysis confirmed the expression of standard MSC surface markers, including CD105, CD73, and CD90, while the cells were negative for CD45, CD34, and HLA‐DR (Figure S2c, Supporting Information), aligning with the established criteria for stem cell identification.

**Figure 2:**
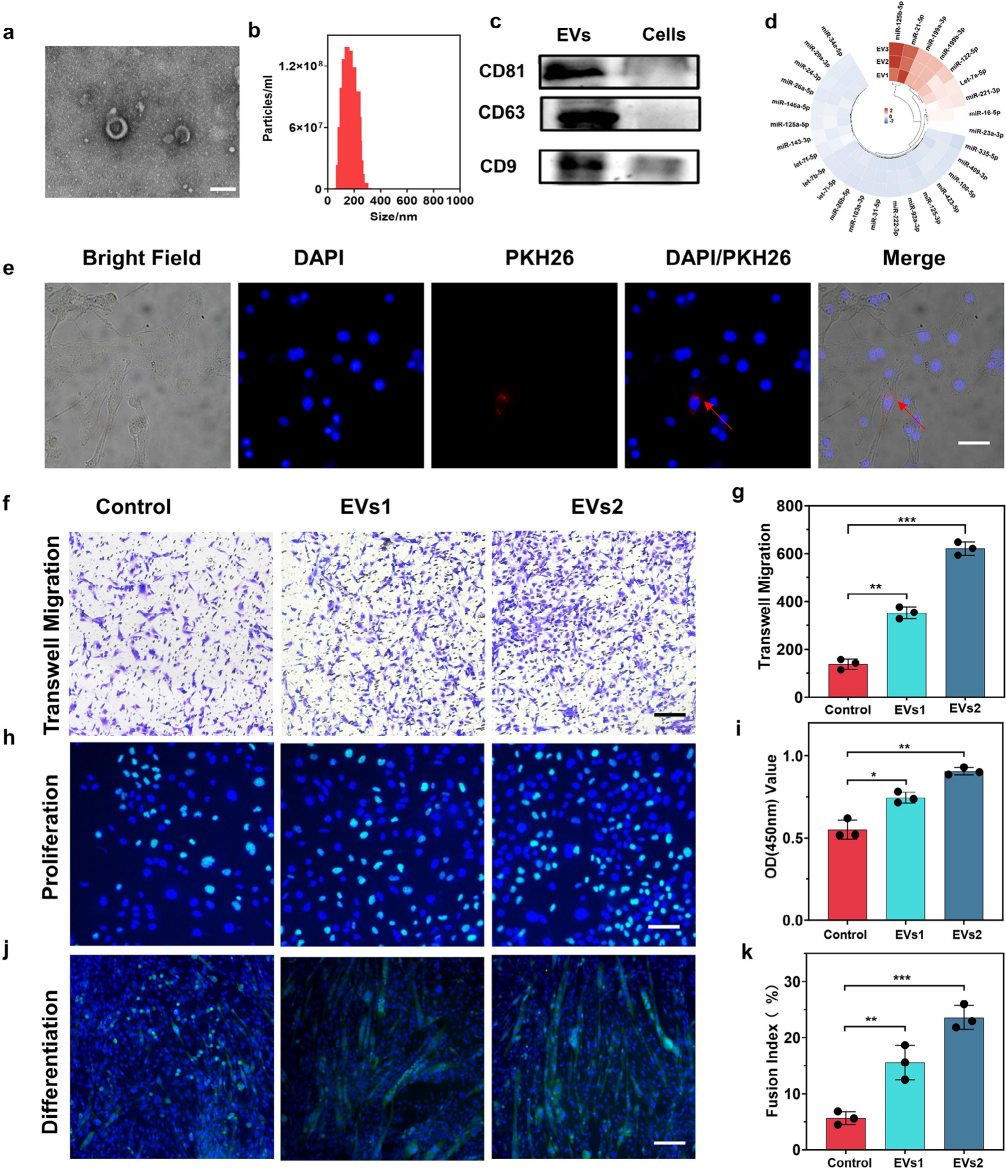
EVs extracted from HUC‐MSC have high biological activity to promote the migration, proliferation, and differentiation of C2C12 cells. a‐b) (a)TEM image and (b) NTA result of EVs, scale bar = 100 nm. c) The typical protein (CD81, CD63, CD9) markers analysis of EVs with HUC‐MSC cells as control. d) Sequencing results of miRNA within EVs. e) Representative bright field image and fluorescence micrograph of PKH‐26 (red) labeled HUC‐MSC‐EVs internalized by C2C12 cells, scale bar = 50 µm. f) Transwell experiment of EVs on C2C12 cell migration, scale bar = 150 µm. g) Analysis of the quantity of C2C12 cell migration influenced by EVs. h) Experiment showing the effect of EVs on cell proliferation, scale bar = 50 µm. i) Cell proliferation assay using CCK8 influenced by EVs. j) Experiment demonstrating the promotion of C2C12 cell differentiation by EVs, scale bar = 100 µm (blue represents DAPI staining, green represents MYOG staining). k) Fusion index of C2C12 cells (the percentage of nuclei within MYOG+ cells containing ≥2 nuclei). **p < 0.01 versus the control; ***p < 0.001 versus the control. Data are presented as the mean ± standard.

We next isolated EVs from HUC‐MSCs using the ultracentrifugation method as previously described.^[^ ^29^, ^37^ ^]^ Characterization of the isolated EVs was performed to confirm their identity and quality. Transmission electron microscopy (TEM) revealed the characteristic cup‐shaped morphology of EVs (**Figure** **2**a), consistent with previous reports. Nanoparticle tracking analysis (NTA) indicated that the majority of EVs were between 80 and 200 nm in diameter, with an average size of 142.5 nm (Figure 2b), matching the expected size range for exosomes. Western blot analysis confirmed the presence of classical EV surface markers, including CD63, CD81, and CD9, while these markers were absent in the original HUC‐MSCs, further validating the successful isolation and enrichment of EVs (Figure 2c)

Subsequently, miRNA sequencing revealed a high abundance of miRNAs in HUC‐MSCs‐EVs (Figure 2d). miRNAs are known to regulate gene expression by binding to the 3′UTR of target genes.^[^ ^50^ ^]^ Target gene prediction using multiple databases indicated that miRNAs enriched in HUC‐MSCs‐EVs are involved in pathways related to cell cycle, DNA replication, insulin resistance, and various signaling cascades (Figure S3a, Supporting Information). Gene Ontology (GO) and Kyoto Encyclopedia of Genes and Genomes (KEGG) analyses further showed that these miRNA‐regulated genes are implicated in processes such as cell migration, proliferation, and differentiation of C2C12 cells (Figure S3b–c, Supporting Information). This well‐established and commonly used mouse myoblast cell line, derived from the muscle tissue of a mouse, was used to validate these findings through additional WB experiments, which confirmed the protein‐level changes associated with these biological processes. Representative protein such as ERK, JNK, p38, and PKA, phosphorylation levels in the energy metabolism and proliferation related signaling pathways enriched with miRNA target genes were detected through WB experiments and quantitatively analyzed (Figure S3d–h, Supporting Information). The results showed that the phosphorylation levels of these representative proteins were significantly increased and correlated with the concentration of EVs, indicating that the addition of EVs can indeed activate signaling pathways related to energy metabolism and proliferation.

The biological effects of HUC‐MSCs‐EVs on C2C12 cells were subsequently evaluated. HUC‐MSCs‐EVs were labeled with PKH‐26 and co‐cultured with C2C12 cells for 24 h. Bright field and fluorescence microscopy confirmed successful EV internalization by C2C12 cells, with red fluorescent EVs localizing around the cell nuclei (Figure 2e). Transwell migration assays demonstrated that HUC‐MSCs‐EVs significantly enhanced the migratory capacity of C2C12 cells, with higher EV concentrations (EVs2: 1 × 10⁹ particles mL^−1^) showing a more pronounced effect than lower concentrations (EVs1: 5 × 10⁸ particles mL^−1^) (Figure 2f,g), suggesting a concentration‐dependent therapeutic effect of HUC‐MSCs‐EVs.

E‐du staining showed a significant increase in cell proliferation in both EV‐treated groups compared to the control, with EVs2 exhibiting a greater effect than EVs1 (Figure 2h). These findings were further supported by CCK8 assays, which confirmed the ability of HUC‐MSCs‐EVs to enhance C2C12 cell proliferation (Figure 2i). Additionally, immunofluorescence analysis of myogenin (MYOG), a key marker of myogenic differentiation, revealed that higher concentrations of EVs positively correlated with increased differentiation of C2C12 cells (Figure 2j,k).

Overall, these results highlight the successful isolation of HUC‐MSC‐derived EVs, which enhance migration, proliferation, and differentiation in muscle cells.

### 2.3 In Vitro Biocompatibility of EVs@UR‐Gel

Next, we evaluated whether the UR‐gel loaded with EVs derived from HUC‐MSCs, referred to as EVs@UR‐gel, exhibits enhanced biological activity. To assess the in vitro biocompatibility of EVs@UR‐gel, we prepared a hydrogel with a concentration of 1 × 10^10^ particles mL^−1^ by incorporating 10 µL of EVs suspension (1 × 10^11^ particles mL^−1^) into 90 µL of UR‐gel. The UR‐gel alone served as the control group, while UR‐gel stimulated with ultrasound at an intensity of 1 MHz for 10 min, and EVs@UR‐gel under the same conditions, constituted the experimental groups.

**Figure 3:**
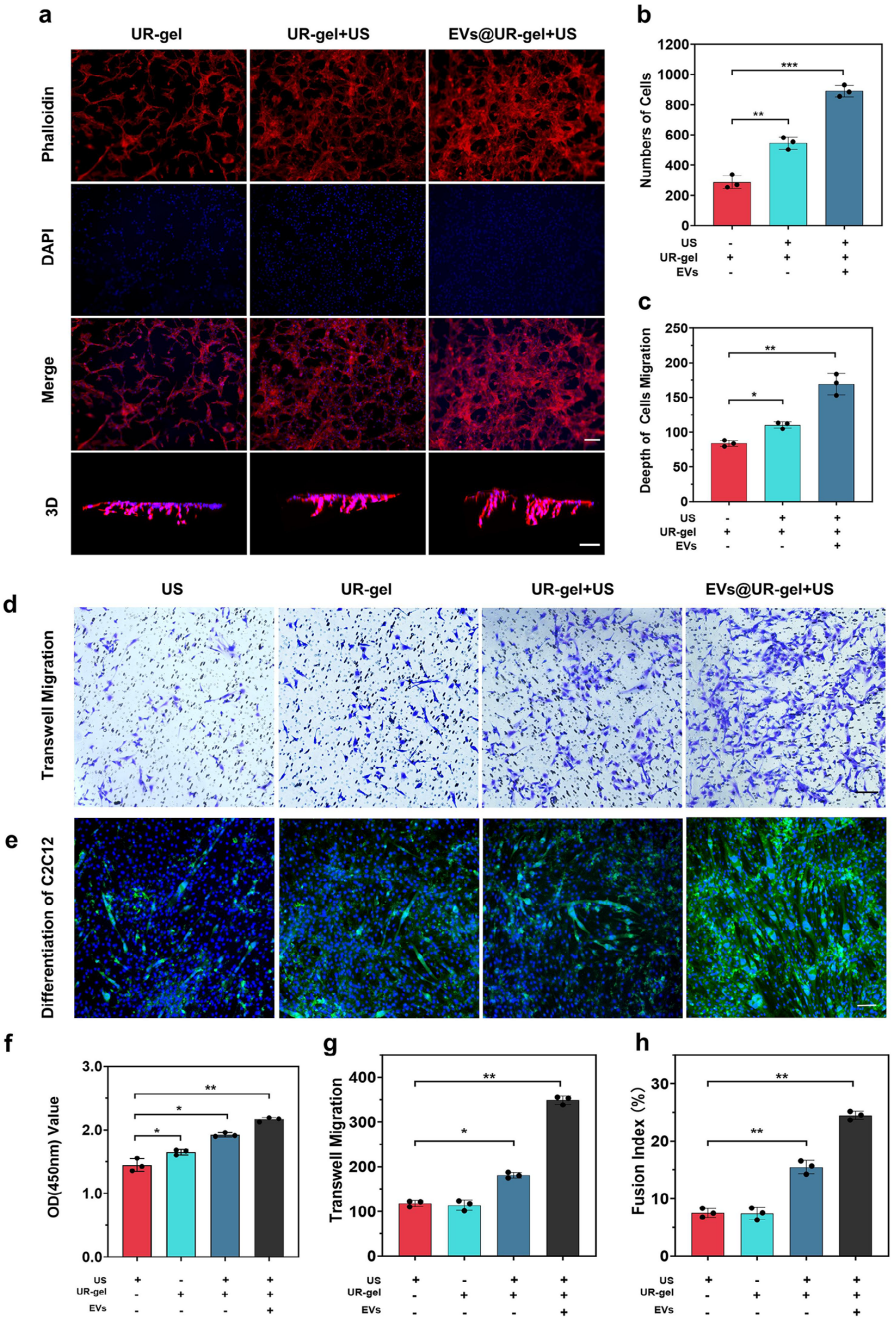
EVs@UR‐gel showed high biological activity in vitro. a) Growth status of C2C12 cells on the scaffold after seeded for 3 days. Phalloidin visually describes the cytoskeleton, DAPI evaluates the number of cells, and 3D displays the depth of cell growth through confocal microscope z‐axis photography, scale bar = 100 µm. b) Number of cells co‐cultured with the scaffold. c) Depth of cell growth on the scaffold. d) Transwell experiment of C2C12 cells with different approaches. e) Differentiation effect of C2C12 cells with different approaches. f) Activity of cells co‐cultured with different approaches. g) Quantity of C2C12 cell migration. h) The fusion index of C2C12 with different approaches. *p < 0.05 versus the US group, **p < 0.01 versus the US group, ***p < 0.001 versus the US group. Data are presented as the mean ± standard.

After co‐culturing C2C12 myoblast cells with the hydrogel for 3 days, Actin/DAPI staining revealed that cells were well spread on the surface of the hydrogel scaffold (**Figure** **3a**). Following ultrasound stimulation, there was a notable increase in both cell number and growth depth within the hydrogel, which was further enhanced by the incorporation of EVs. The quantitative analysis of numbers of cells and depth of cells migration (Figure 3b–c), demonstrated significantly enhanced cell proliferation post‐ultrasound treatment. The addition of EVs to the gel further augmented this proliferative capacity. Specifically, compared to the gel‐only group and the ultrasound‐treated gel group, the ultrasound‐stimulated EVs@UR‐gel group exhibited an increase in nuclear count from 287 ± 36 cells/field to 891 ± 31 cells/field, and an increase in cell growth depth within the hydrogel from 90.5 ± 10 nm to 169.3 ± 12.7 nm.

We next evaluated the impact of EVs@UR‐gel on C2C12 cell migration and differentiation. Cell migration was significantly enhanced in the UR‐gel group after ultrasound stimulation, with a more pronounced effect observed in the EVs@UR‐gel group (Figure 3d). In addition, differentiation experiments demonstrated that MyoG immunofluorescence staining indicated a marked enhancement in C2C12 cell differentiation when treated with EVs@UR‐gel and stimulated with ultrasound (Figure 3e; Figure S4, Supporting Information). Quantitative analysis CCK‐8, transwell, and fusion index confirmed that EVs@UR‐gel significantly outperformed both the UR‐gel and ultrasound‐treated UR‐gel groups in promoting cell proliferation, migration, and differentiation (Figure 3f–h).

These findings suggest that ultrasound stimulation induces structural modifications in the UR‐gel, increasing its porosity and providing additional space for C2C12 cell infiltration and growth. Furthermore, the release of encapsulated EVs post‐ultrasound stimulation facilitates cell penetration into deeper regions of the hydrogel. The significant differences observed between the UR‐gel and EVs@UR‐gel groups highlight the protective role of the hydrogel matrix in maintaining the biological activity of EVs. Notably, even after 10 min of ultrasound stimulation at 1 MHz, the biological efficacy of the EVs remained intact, underscoring the effectiveness of EVs@UR‐gel in promoting cell proliferation, migration, and differentiation.

### 2.4 Anti‐Inflammatory and Antioxidant Effects of UR‐Gel

In the early stages of denervated muscle atrophy, muscle fibers lose voluntary contractile function and experience fibrillation, leading to blood stasis and the development of a hypoxic microenvironment.^[^ ^39^ ^]^ Over time, this hypoxia promotes the infiltration of inflammatory cells, initiating a vicious cycle that accelerates muscle degradation.^[^ ^38^, ^40^ ^]^ Current research has confirmed that oxidative stress is closely related to the damage of skeletal muscle cells, and the clearance of oxygen free radicals can effectively protect skeletal muscle cells.^[^ ^51^, ^52^ ^]^ In this context, the capacity of carrier materials to neutralize free radicals is crucial for effective therapeutic intervention. Therefore, the anti‐inflammatory and antioxidant properties of UR‐gel were assessed through a series of targeted assays.

**Figure 4:**
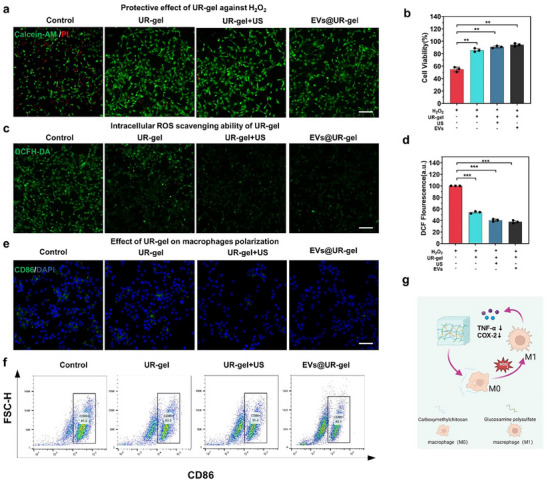
UR‐gel has anti‐inflammatory and antioxidant abilities. a) Live/dead staining of C2C12 cells, the blank group was set as control, the green fluorescence represented living cells, and the red fluorescence represented dead cells, indicating that EVs@UR‐gel had a protective effect on C2C12 cell death induced by H_2_O_2_, scale bar = 100 µm. b) Cell viability assay after co‐cultured with H_2_O_2_ for 24 h. c) Intracellular ROS scavenging ability of EVs@UR‐gel in H_2_O_2_‐induced C2C12 using DCFH‐DA as a fluorescent ROS probe, scale bar = 100 µm. d) Intensity of ROS fluorescence. e) After being incubated with 100 ng mL^−1^ LPS for 24 h beforehand, RAW 264.7 cells treated with PBS, UR‐gel, UR‐gel+US and EVs@ UR‐gel, and analyzed by immunofluorescent staining of CD86, scale bar = 100 µm. f) flow cytometry of CD86 expression. g) Schematic diagram of the gel immune regulation process. (Created with bioRender.com). **p < 0.01 versus the control. Data are presented as the mean ± standard.

To assess the protective capacity of EVs@UR‐gel against oxidative damage, Calcein‐AM/PI staining was performed following H_2_O_2_ treatment to induce ROS‐mediated stress. The addition of EVs@UR‐gel increased the number of live cells, and a slight further increase was observed after ultrasound treatment (**Figure** **4**a; Figure S5a, Supporting Information). The CCK‐8 assay results demonstrated enhanced cell viability in the presence of EVs@UR‐gel, regardless of ultrasound exposure (Figure 4b), indicating a protective effect against ROS. The ultrasound treatment did not significantly impact ROS resistance, suggesting that EVs@UR‐gel alone offers substantial protection.

To quantify intracellular ROS levels, 2′,7′‐dichlorofluorescin diacetate (DCFH‐DA) staining was used. The fluorescence intensity, indicative of ROS levels, was reduced in the EVs@UR‐gel group, demonstrating a downregulation of H_2_O_2_‐induced ROS (Figure 4c,d). The ability of cells to resist ROS was further enhanced after ultrasound treatment, likely due to structural changes in the hydrogel induced by ultrasound, which may promote the internalization of hydrogel components by cells, enhancing their intracellular anti‐ROS capacity.

Given the strong association between inflammation and M1 macrophage polarization, the anti‐inflammatory effects of EVs@UR‐gel were investigated in RAW264.7 macrophages stimulated with lipopolysaccharide (LPS). A significant reduction in CD86 expression (characteristic of M1 macrophages) was observed, indicating a reduction in M1 polarization (Figure 4e; Figure S5b, Supporting Information). Combined with the above results, the application of ultrasound further reduced intracellular ROS levels, restored macrophages to the M0 (non‐polarized) state, and promoted the adoption of an elongated spindle morphology, typical of M2 macrophages, which are associated with anti‐inflammatory activity. Flow cytometry analysis following anti‐CD86 fluorescence labeling further confirmed the reduction in M1 macrophage polarization. The proportions of M1 macrophages in the different treatment groups were 81.2% (control), 63.3% (UR‐gel), 51.4% (ultrasound‐stimulated UR‐gel), and 43.1% (EVs@UR‐gel) (Figure 4f).

Taken together, these results underscore the hydrogel's potent antioxidant and anti‐inflammatory effects (Figure 4g), make it a promising option for early therapeutic intervention in denervated muscle atrophy.

### 2.5 Effects of EVs@UR‐Gel on Motor Function Recovery

**Figure 5:**
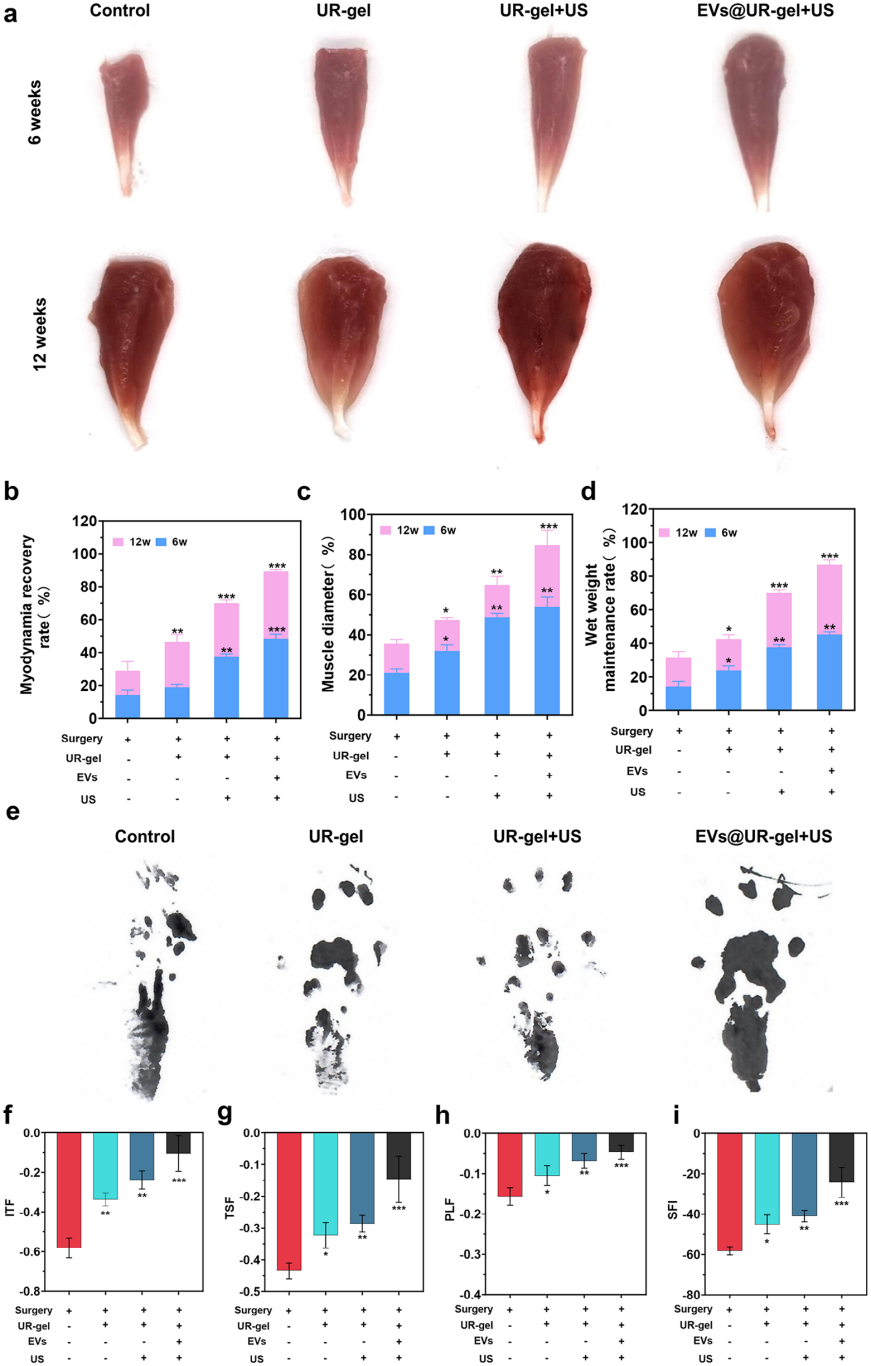
EVs@UR‐gel promotes the recovery of motor function. a) Photograph of tibialis anterior muscle from rats after treatment with various strategies for 6 weeks and 12 weeks in different groups. b) Proportion of maximal muscle tension retained in the tibialis anterior muscle of rats. c Diameter of the tibialis anterior muscle in rats. d) Wet weight of the tibialis anterior muscle in rats. e) SFI analysis of rats at 12 weeks. f–g) Numerical analysis related to SFI in rats. *p < 0.05 versus the control; **p < 0.01 versus the control; ***p < 0.001 versus the control. Data are presented as the mean ± standard.

To evaluate the reparative effects of EVs@UR‐gel on denervated muscle atrophy, the hydrogel was injected into the tibialis anterior muscle of rats during model establishment. Gross examination revealed that the tibialis anterior muscle in the EVs@UR‐gel group was thicker compared to other groups (**Figure** **5**a). After 6 weeks of denervation, the EVs@UR‐gel group exhibited a greater residual muscle circumference, higher passive muscle force, and increased muscle wet weight compared to the control groups (Figure 5b–d). At 6 weeks post nerve repair (week 12), the maximum muscle strength, muscle circumference, and muscle wet weight in the EVs@UR‐gel group were restored to 89.53 ± 0.96%, 76.02 ± 7.49%, and 88.0 ± 2.65% of the healthy state, respectively. The intervention with EVs@UR‐gel reduced the rate of muscle atrophy and preserved muscle strength, enhancing the muscle's recovery potential following denervation.

To further investigate the protective effects of EVs@UR‐gel on motor function after denervation and repair, gait analysis was performed at week 12. Motor function was assessed using the Sciatic Functional Index (SFI), where 0 represents normal function and ‐100 indicates complete loss of motor function. In the assessment of normal motor function, values of PLF, TSF, and ITF were 0. Results showed that at week 12, except for the sham surgery group, all motor function indicators and the comprehensive SFI in the EVs@UR‐gel group were significantly higher than in other groups (Figure 5e–i).

In order to verify the temporal distribution of EVs in vivo, we conducted in vitro fluorescence detection on the modeled rats. We stained EVs with EVs DIR dye and then injected EVs into the body using PBS and UR‐gel as carriers. The distribution of EVs was obtained through in vitro fluorescence detection and subjected to Radiant efficiency quantitative analysis. The results showed that direct injection of EVs was quickly absorbed, and the testing was basically consumed within 3 days. And UR‐gel can effectively retain EVs in vivo, and the release rate significantly increases after US stimulation (Figure S6, Supporting Information). This indicates that the use of UR‐gel as a carrier for EVs achieved controlled release.

Thus, these findings suggest that the EVs@UR‐gel hydrogel effectively preserves motor function in target muscles following denervation.

### 2.6 Immunofluorescence Staining Results and Gross Specimens

**Figure 6:**
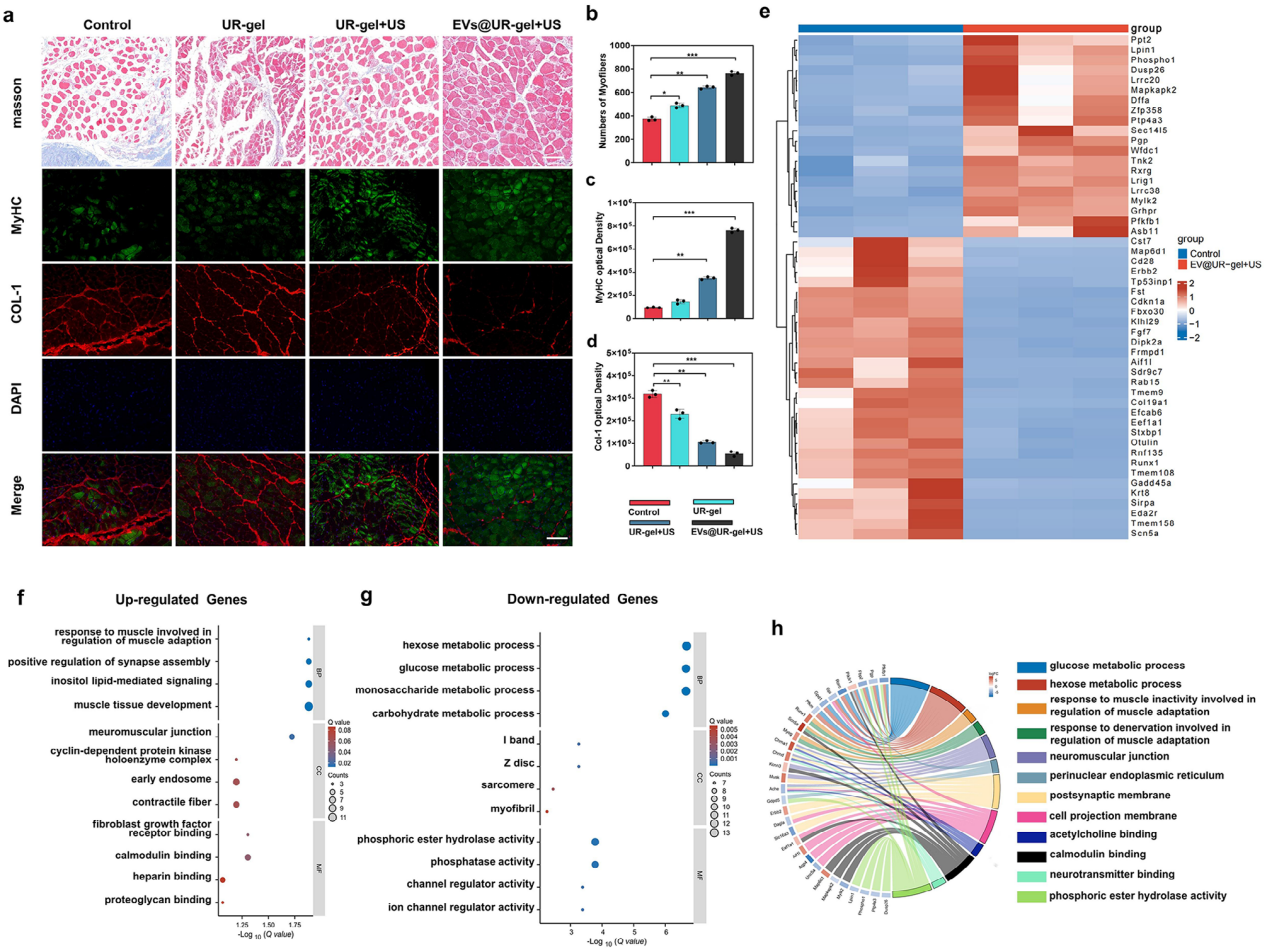
Effects of EVs@UR‐gel on muscle regeneration and type I collagen deposition in target muscle in vivo. a) after treatment for 6 weeks, the tissue sections stained with Masson's trichrome and immunofluorescence (green: MyHC immunofluorescence staining; red: Col‐1 immunofluorescence staining; blue: DAPI staining; merged image), EVs@UR‐gel can promote the regeneration of MyHC and inhibit the deposition of COL‐1, scale bar = 100 µm. b‐d) The semi‐quantification of (b) numbers of myofibers, (c) MyHC and (d) Col‐1 immunofluorescence signal intensity. e‐f) Go analysis of changes in transcriptome profile of target muscle treated by EVs@UR‐gel+US. group and the control group ‐in (e) up‐ and (f) down‐ regulated genes. g) Differentially expressed genes (DEGs) analysis between the EVs@UR‐gel+US. group and the control group. h) GO Chord plot of interested GO terms and the corresponding DEGs.

Tissue sections of the tibialis anterior muscle at the 6th week were analyzed using Masson's staining and immunofluorescence staining to evaluate the protective effects of EVs@UR‐gel on denervated muscles. Masson's staining revealed that muscle fibers in the EVs@UR‐gel group maintained structural integrity, exhibited a tightly organized arrangement, and had reduced collagen fiber thickness with a more orderly extracellular matrix, in contrast to the other groups (**Figure** **6a**). Muscle atrophy was significantly mitigated, and fibrosis was less pronounced in the EVs@UR‐gel group.

Further immunofluorescence staining using antibodies against MyHC (myosin heavy chain) and Col‐1 (collagen type I) provided insights into the molecular effects of the treatment. MyHC, a principal contractile protein essential for muscle contraction, showed increased fluorescence intensity and improved fiber morphology in the EVs@UR‐gel group, indicating better muscle preservation and regeneration compared to other groups. In contrast, Col‐1, a key marker of muscle fibrosis, showed reduced fluorescence intensity in the EVs@UR‐gel group. The sequential increase in MyHC fluorescence intensity—from the control group to the UR‐gel group, ultrasound‐treated UR‐gel group, and EVs@UR‐gel group—highlighted the enhanced reparative effects of the EV‐loaded hydrogel. The decrease in Col‐1 fluorescence across these groups further supports the anti‐fibrotic effects of the treatment. Quantitative analysis confirmed these trends (Figure 6b–d), underscoring the ability of EVs@UR‐gel to promote muscle fiber regeneration while inhibiting fibrosis.

In order to investigate the therapeutic effects of EVs encapsulated within UR‐gel on denervated muscle atrophy at the gene level. we conducted mRNA sequencing of tibial anterior muscle specimen of rats at six weeks postoperatively to elucidate the differential gene expression profiles between the control group and the EVs@UR‐gel treated group. This comparative analysis revealed significant transcriptional changes, with 560 genes being upregulated and 315 genes being downregulated in the EVs@UR‐gel treated group as compared to the control. These findings provide insights into the molecular mechanisms by which EVs@UR‐gel modulates gene expression, potentially leading to therapeutic benefits in muscle regeneration and function preservation.(Figure S7, Supporting Information). GO and KEGG pathway analysis (Figure 6e–h) revealed significant enrichment in pathways related to muscle development, muscle contraction, anti‐inflammatory responses, and metabolic regulation. These findings indicate that the application of EVs@UR‐gel supports the functional recovery of denervated muscles and stabilizes the internal cellular environment at a genetic level.

## 3 Discussion

Denervated muscle atrophy is a common complication following trauma and severely impacts patients' quality of life.^[^ ^3^ ^]^ Currently, the treatment of denervated muscle atrophy primarily focuses on nerve repair. However, due to delayed treatment and slow peripheral nerve axonal growth, skeletal muscle inevitably undergoes functional degradation before the axons reach the target organs. Skeletal muscle cells are highly differentiated cells that have lost their proliferative capacity, and their regeneration primarily depends on the satellite cells attached to the muscle fibers.^[^ ^53^, ^54^ ^]^ After prolonged loss of neural stimulation, the atrophy of skeletal muscle cells and the depletion of satellite cells in the target muscle are the major causes of the muscle's loss of regenerative capacity.^[^ ^55^ ^]^ In recent years, with the deepening understanding of its pathogenesis, researchers have progressively revealed the pathological and physiological processes that occur after denervation.^[^ ^56^ ^]^ Through gene expression microarray analysis and tissue section examination, studies have shown that the histological and molecular biological changes in the target muscle tissue occur in a characteristic pattern, which becomes more pronounced as the denervation duration increases.^[^ ^57^, ^58^, ^59^ ^]^ During the early stages of denervation, oxidative stress (involving p53 signaling, PPAR signaling, HIF‐1 signaling) and inflammation‐related pathways (such as TNF signaling, NOD‐like receptor signaling, TGF‐β signaling) are widely activated. In the later stages, apoptotic and cell senescence‐associated pathways are predominantly activated, while metabolic pathways, oxidative phosphorylation, the citric acid cycle, and glycolysis‐related signaling pathways are significantly suppressed.^[^ ^59^ ^]^ Considering these characteristics, an effective treatment for denervated muscle atrophy should possess the following features: (1) in the early stage, the ability to remove reactive oxygen species (ROS), regulate pH, and stabilize the local microenvironment; (2) in the later stage, the ability to release therapeutic agents in a controlled manner to slow skeletal muscle atrophy and promote the proliferation, migration, and differentiation of satellite cells.^[^ ^60^, ^61^ ^]^ Based on this concept, we developed a pH/ultrasound dual‐responsive injectable hydrogel, which, while treating denervated muscle atrophy, also serves as a carrier for EVs to achieve controlled release. Additionally, we isolated EVs from HUC‐MSCs and validated their ability to effectively promote the migration, proliferation, and differentiation of myogenic cells. In vivo experimental results show that HUC‐MSC‐derived EVs can be released in a controlled manner as needed and effectively maintain the function and morphology of denervated skeletal muscle.

Carboxymethyl chitosan (CMCS) is an important derivative of chitosan that retains the high biocompatibility and anti‐inflammatory properties of the original chitosan. Additionally, its surface carries numerous amino groups, allowing its surface charge to switch between positive and negative as the environmental pH changes.^[^ ^62^ ^]^ Research by Kalliola et al. has demonstrated that chitosan‐based hydrogels exhibit pH‐responsive properties, suggesting that CMCS holds great potential for treating denervated muscle atrophy.^[^ ^63^ ^]^ However, a single type of response is insufficient to achieve controlled material properties necessary for treating denervated muscle atrophy. Among the common regulatory approaches, ultrasound has been widely applied in smart composite biomaterials.^[^ ^64^ ^]^ Unlike visible light, magnetic fields, or electric fields, ultrasound is an inaudible periodic mechanical wave that propagates effectively in complex media, precisely targeting specific spatial locations and delivering energy to deeper tissues.^[^ ^65^, ^66^ ^]^ Previous studies have shown that hydrogels, with their high water content and soft crosslinked polymer network structure, can mimic the natural aqueous environment of the human body. This provides a cellular environment similar to the natural extracellular matrix, which is conducive to cell growth and proliferation, giving them an inherent advantage in muscle tissue repair.^[^ ^67^ ^]^ In this study, we developed a pH/ultrasound dual‐responsive injectable hydrogel by crosslinking aldehyde‐modified chondroitin sulfate (obtained by oxidation with sodium periodate) with CMCS and cystamine dihydrochloride through the Schiff base reaction between the amino and aldehyde groups. Subsequent FTIR spectra confirmed the complete crosslinking and successful formation of the hydrogel, as evidenced by the disappearance of the aldehyde peak at 1736.5 cm^−1^. SEM images revealed distinct porous structures in the hydrogel, and ultrasound stimulation resulted in significant changes in the release and degradation rates of the hydrogel. Thermal imaging results indicated that these changes were not due to the thermal effect of ultrasound, but likely resulted from the energy and internal ionic bond transformations (such as hydrogen bonds and disulfide bonds) facilitated by acoustic radiation force (AFR) and cavitation.^[^ ^65^, ^66^ ^]^ These findings suggest that UR‐gel, as a pH/ultrasound dual‐responsive injectable hydrogel, possesses early anti‐inflammatory and antioxidant properties, and the ability for controlled drug release in the later stages, making it an excellent carrier for treating denervated muscle atrophy.

EVs derived from HUC‐MSCs have the advantages of high biological activity, strong stability, easy accessibility, and abundant sources. These properties give them potential applications in the treatment of musculoskeletal injuries, particularly those in the locomotor system. Studies have shown that exosomes exhibit functions similar to those of the parental cells. Our previous research demonstrated that HUC‐MSCs‐EVs through their miRNA‐23a‐3p content, promote chondrocyte migration, proliferation, and differentiation by activating the PTEN/AKT signaling pathway. Recent studies have also shown that overexpression of miR‐23a in muscle can prevent diabetes‐induced muscle wasting.^[^ ^68^ ^]^ However, the pathophysiology of neurogenic muscle atrophy involves the suppression or downregulation of multiple signaling pathways, and a single signaling pathway cannot fully explain the mechanisms by which exosomes exert their effects. In this study, we conducted sequencing analysis and target gene prediction on exosomes isolated by differential centrifugation. The results indicated that miRNA target genes enriched in HUC‐MSCs‐EVs are involved in metabolic pathways, oxidative phosphorylation, the citric acid cycle, and glycolysis‐related signaling pathways. Upon co‐culturing with C2C12 cells in vitro, the exosomes were successfully internalized by C2C12 cells, further promoting their migration, proliferation, and differentiation. Western blotting analysis showed a significant increase in the phosphorylation of MAPK, ERK, and cAMP proteins, suggesting that the activation of multiple signaling pathways is triggered following exosome internalization. In conclusion, HUC‐MSCs‐EVs may provide a promising new therapeutic approach for the treatment of neurogenic muscle atrophy, based on the findings of this study.

Research has confirmed that adopting different therapeutic strategies according to the various stages of a disease results in significantly better treatment outcomes compared to single therapeutic approaches.^[^ ^69^ ^]^ Li et al. designed a dual EV delivery system with spatiotemporal controlled release based on the pathogenesis of osteoarthritis, which demonstrated a notably superior therapeutic effect on osteoarthritis compared to traditional treatment methods.^[^ ^70^ ^]^ The system proposed in this study, which involves ultrasound‐controlled release of EVs, follows the same principle, but with more precise stage‐specific release. However, a critical concern regarding ultrasound‐driven responsive hydrogels is that ultrasound, being a mechanical stimulus, may potentially damage EVs. To address this issue, we initially employed low‐intensity ultrasound in the design of the hydrogel. Furthermore, to simultaneously assess the impact of ultrasound on the hydrogel and its protective effect on EVs, we cultivated cells in the system both before and after ultrasound stimulation and observed the depth of cell growth. The results indicated that after ultrasound intervention, cell growth depth was significantly enhanced. Notably, in the EVs@UR‐gel group, cells grew deeper into the hydrogel. This experiment suggests that UR‐gel undergoes structural changes upon ultrasound stimulation and can release biologically active EVs, which in turn promote cell proliferation and migration, encouraging deeper cellular penetration into the hydrogel. In vivo experimental results revealed that the EVs@UR‐gel system effectively maintained key functional indicators of muscle, such as maximum muscle strength, muscle wet weight, and muscle circumference, in the tibialis anterior muscle following denervation. Both footprint analysis and SFI showed significant recovery. Footprint analysis provided a direct understanding of the animal's gait, while SFI offered a quantitative assessment tool. Combined, these two methods allowed for a more comprehensive evaluation of muscle function and the recovery of nerve damage in rats.^[^ ^71^ ^]^ Histological staining further illustrated that the EVs@UR‐gel system offered protective effects on the structure of the tibialis anterior muscle after denervation, significantly reducing the production of Type I collagen. These findings align with the results of previous studies, which suggest that EVs can effectively reduce muscle fibrosis and maintain the content of muscle functional proteins such as MyHC.

In summary, this injectable hydrogel system, designed based on the pathophysiological processes of the disease, incorporates pH and ultrasound dual‐response characteristics and is loaded with HUC‐MSC‐EVs. It not only allows for controlled release of EVs in response to disease‐specific needs but also provides anti‐inflammatory and antioxidant effects. The system effectively reduces muscle fibrosis, maintains muscle structure, and facilitates the recovery of muscle function following nerve reconnection during the progression of neurogenic muscle atrophy.

## 4 Conclusion

We discovered that the miRNA target genes in HUC‐MSCs‐EVs are associated with cellular energy metabolism and proliferation pathways, suggesting the potential of these genes in preventing denervated muscle atrophy. To efficiently load, protect, and deliver HUC‐MSCs‐EVs, we developed an ultrasound/pH dual‐responsive anti‐inflammatory injectable hydrogel. This hydrogel exhibited desirable anti‐inflammatory properties and ultrasound controllability. At the early stage of the disease, the hydrogel's pH responsiveness was achieved. The anti‐inflammatory and ROS‐neutralizing effects of the hydrogel were observed at the initial stage of the disease. At the subsequent stage of muscle atrophy, ultrasound intervention enabled the modifiable release of EVs, which aided in maintaining muscle function and structure after neuromuscular atrophy and enhanced the therapeutic efficacy of HUC‐MSCs‐EVs. Overall, the EVs@UR‐gel demonstrates significant potential in preventing denervated muscle atrophy and preserving muscle function.

## 5 Experimental Section

### Synthesis of Oxidized Chondroitin Sulfate (OCS)

OCS was synthesized by an oxidation reaction using NaIO4(Aladdin, S104093, China). First, 1.0 g of chondroitin sulfate (CS) (Aladdin, C960198, China) was dissolved in 20 mL distilled water. Then, 700 mg of NaIO_4_ was dissolved in 20 mL distilled water. After dissolving completely, two solutions were mixed and stirred for 6 h in the dark at room temperature. Subsequently, 1 mL of ethylene glycol was added into the reaction system to terminate the oxidation reaction for 1 h. And then, the solution was dialyzed with distilled water for 3 days and freeze‐dried, resulting in a white solid sample. OCS was stored at room temperature and protected away from light.

### Fabrication of pH/Ultrasound Dual‐Responsive Hydrogel

The pH/ultrasound dual‐responsive hydrogel was prepared based on the principle of the Schiff base reaction. In brief, carboxymethyl chitosan (CMCS, 1.0 g) (Aladdin, C304739, China) was dissolved in 50 mL of deionized water with stirring to completely dissolved. OCS (500 mg) was dissolved in 5 mL of deionized water to obtain 100 mg mL^−1^ of OCS. Cystamine dihydrochloride (Macklin, 768 761, China) was dissolved in deionized water to 1.0 mol L^−1^. And then, CMCS, OCS, and cystamine dihydrochloride solutions were mixed at a volume ratio of 8:1:1 at room temperature with slight stirring. The pH/ultrasound dual‐responsive hydrogel was gradually solidified and fabricated.

### Fourier Transform Infrared Spectra (FTIR) Measurement

FTIR data were recorded using a Bruker Equinox 55 spectrometer at frequencies ranging from 400 to 4000 cm^−1^ and resolution of 0.5 cm^−1^. Samples were powdered and mixed with dried KBr powder and pressed into pellet form.

### Morphology of Hydrogels

The morphologies of hydrogels were analyzed by scanning electron microscopy (SEM, Zeiss microscope). Samples were washed for three time with deionized water and frozen in −20 °C. Then the samples were freeze‐dried to obtain anhydrous samples. Both hydrogels before and after ultrasound treatment were observed by SEM.

### Rheology Test of Hydrogels

The hydrogels were performed on HAKKE rheometer to study the rheological property. The viscoelastic properties of the hydrogels were measured by performing strain sweep experiments in the oscillation mode. The frequency was set at 1 Hz, and the storage modulus (*G*′) and loss modulus (*G*″) values were recorded by sweeping tests changing the strain from 0.1% to 1000%. The hydrogels were divided into two groups, one untreated and the other sonicated by ultrasound diagnostic equipment (1.0 mW cm^−2^, 10 min).

### Swelling Behavior Studies of Hydrogels

Anhydrous hydrogels after freeze‐drying were weighed (W_D_), and then stored in PBS buffer to allow water uptake. The swollen hydrogels were extracted and weighed (W_S_) after wiping to remove excessive water at different time points, and the W_S_/W_D_ ratio was calculated. The experiment was measured continuously for 10 h to fully analyze the swelling behavior of the hydrogels.

### Biodegradation Behavior of Hydrogel

Anhydrous hydrogels after freeze‐drying were weighed (W_0_) and immersed in PBS buffer (pH = 7.4) with 0.4 mg mL^−1^ of lysozyme (1 × 10^5^ U mg) at 37 °C. The hydrogels were taken out and weighed (W_s_) at different times. The experiment was measured continuously for 6 d to fully analyze the biodegradation behavior of the hydrogels. The degradation rates were calculated using the following formula: The degradation rate = (1 – W_s_/W_0_) × 100%.

### In Vitro Drug Release

The in *vitro* drug release behavior of the pH/ultrasound dual‐responsive hydrogel was investigated using doxorubicin (Aladdin, D107159, China) as the model drug. First, a range of concentrations of DOX from 0 to 25 µg mL^−1^ were prepared. And the absorbance was measured by UV‐visible spectroscopy (INSEA, China). The peak absorbance at 465 nm was recorded to fit the calibration curve of MP. And then, 20 µL of DOX solution (1.0 mg mL^−1^) was added into the 980 µL of the uncross‐linked hydrogel solution to prepare DOX‐loaded hydrogel (20 µg DOX per 1.0 mL hydrogel). The hydrogel of free releasing was set as control. The DOX‐loaded hydrogels were immersed in 5.0 mL PBS buffer (pH 7.4) to study release behavior. And hydrogels of ultrasound treatment, pH treatment (immersing into pH 6.0 PBS buffer), ultrasound + pH treatment were set as experimental group. The ultrasound treatment was set at 60th, 120th, 180th, and 300th min, and each ultrasound treatment (1MHZ, 45 W) lasted 10 min. The absorbance of DOX was measured by UV‐visible spectroscopy and the released percentages were calculated by the fitted calibration curve.

### Cell Acquisition and Culture

As previously described,^[^ ^29^, ^37^ ^]^ human umbilical cord mesenchymal stem cells (Cyagen, HUXUC‐01001, China) were purchased from Cyagen Biotechnology Company. The HUC‐MSCs were cultured in Ultra culture medium (Lonza, USA, 12–725 F) contained serum analogue Ultroser G (Life Science, USA, 259 509). The multilineage differentiation potential of HUC‐MSCs was verified by inducing osteogenic, chondrogenic, and adipogenic differentiation using differentiation media. Surface markers of HUC‐MSCs (CD105, CD73, CD90, CD45, CD34, HLA‐DR) (TBD, TBD‐MSCFC, China) were detected using flow cytometry. C2C12 myoblast cell line was purchased from Procella (Procella, CL‐0044, China). The cells were cultured in DMEM (Gibico, C11995500BT, USA) supplemented with 10% fetal bovine serum (Gibico, A3160901, USA), 100 IU mL^−1^ penicillin, and 100 µg mL^−1^ streptomycin. When the myoblasts reached 70%–80% confluence, they were induced to differentiate into myotubes using DMEM supplemented with 2% horse serum (Gibico, 16 050 122, USA), 100 IU mL^−1^ penicillin, and 100 µg mL^−1^ streptomycin. RAW cells (RAW 264.7, a murine‐derived macrophage cell line) were obtained from the cell bank of the Chinese Academy of Sciences Typical Culture Preservation Committee, and cultured with DMEM (Gibico, C11995500BT, USA) supplemented with 10% fetal bovine serum (Gibico, A3160901, USA), 100 IU mL^−1^ penicillin, and 100 µg mL^−1^ streptomycin.

### EVs Isolation and Characterization

The supernatant from HUC‐MSCs culture was collected for EVs isolation using ultracentrifugation. The specific steps were as follows: First, HUC‐MSCs were cultured in three T75 flasks until they reached 70–80% confluence. The cells were then cultured in Ultra culture medium (Lonza, 12–725 F, USA) containing 10% EVs‐free FBS (System Biosciences, 50A‐1, USA) for a period of 72 h to allow for the accumulation of EVs in the conditioned medium. Then, the collected cell supernatant was centrifuged at 300 g, 4 °C for 10 min to remove cell debris. The supernatant was then centrifuged at 2000 g, 4 °C for 10 min to remove dead cell debris. Subsequently, the supernatant was centrifuged at 10 000 g, 4 °C for 30 min to remove cellular debris. The resulting supernatant was filtered through a 0.22 µm filter and transferred to ultracentrifuge tubes. Ultracentrifugation was performed by using a Type 70 Ti Fixed Angle Rotor at 100 000 g (Beckman Coulter, USA) for 70 min at 4 °C. The pellet obtained after centrifugation was resuspended in 1 mL PBS.^[^ ^72^ ^]^ Next, Western blotting was then performed to detect the surface markers CD63, CD81, and CD9 of the EVs by using anti‐CD63(SBI, EXOAB‐CD63A‐1, USA, 1:1000), anti‐CD81(SBI, EXOAB‐CD81A‐1, USA, 1:1000), anti‐CD9(SBI, EXOAB‐CD9A‐1, USA,1:1000).

### Nanoparticle Tracking Analysis (NTA)

The NTA was employed to determine the particle size and concentration of EVs. The analysis was conducted using the Zetaview PMX 110 instrument (Particle Metrix, Meerbusch, Germany) equipped with the corresponding software ZetaView 8.04.02. Prior to analysis, EVs were pre‐diluted with 1× PBS to a dilution factor of 1:1000 and then injected into the sample‐carrier cell. The NTA measurements were recorded and analyzed at 11 different positions to ensure accuracy and consistency. The ZetaView system was calibrated using 110 nm polystyrene particles to ensure the reliability of the measurements. Throughout the procedure, the temperature was meticulously maintained at 27.65 °C.

### PKH‐26 Staining and Cellular Uptake Assay

The PKH‐26 staining kit (Solarbio, D0030, China) was employed to label the EVs. The brief procedure, as provided by the manufacturer, is outlined below: The previously isolated EVs were diluted with Dilution C solution, followed by the addition of 4 µL of PKH‐26 dye solution. The mixture was then incubated at room temperature in the dark for 5 min. Subsequently, the staining reaction was terminated by adding 500 µL of 1% BSA solution. The labeled EVs were pelleted by centrifugation at 1 000 000 g for 70 min at 4 °C, followed by resuspension in 200 µL of cold PBS. C2C12 cells were co‐cultured with the labeled EVs for 24 h. The cells were then fixed with 4% paraformaldehyde, stained with DAPI for 5 min to label the cell nuclei, and observed under a fluorescence microscope to visualize the stained cells.

### miRNA Sequencing Analysis

Total RNA was extracted from the tissue using TRIzol Reagent according to the manufacturer's instructions. Then RNA quality was determined by 5300 Bioanalyser (Agilent) and quantified using the ND‐2000 (NanoDrop Technologies). Only high‐quality RNA samples were used to construct sequencing library. RNA purification, reverse transcription, library construction and sequencing were performed at Shanghai Majorbio Bio‐pharm Biotechnology Co., Ltd. (Shanghai, China) according to the manufacturer's instructions (Illumina, San Diego, CA).

### miRNA Identification, Differential Expression Analysis, and Target Gene Predictions

The identification and differential expression analysis of miRNAs were conducted using a comprehensive approach. Initially, small RNA tags were mapped to identify known miRNAs with the miRBase22.0 database (http://www.mirbase.org/). Subsequently, the remaining tags were aligned with the Rfam and Repbase databases to exclude ribosomal RNA (rRNA), transfer RNA (tRNA), small nuclear RNA (snRNA), small nucleolar RNA (snoRNA), and other non‐coding RNAs (ncRNA) and repeats. Novel miRNAs were predicted and identified using mirdeep2 software based on their genomic positions and hairpin structures. The expression levels of each miRNA were calculated using the transcripts per million reads (TPM) method. Differential expression analysis was performed using DESeq2 or DEGseq, with significantly differentially expressed genes (DEGs) defined as those with |log2FC| ≥ 1 and FDR < 0.05 (DESeq2) or FDR < 0.001 (DEGseq). For target gene predictions of miRNAs, miRanda was used for animal miRNAs, and psRobot was used for plant miRNAs. The predicted target genes were annotated with the GO (http://www.geneontology.org/) and KEGG (http://www.genome.jp/kegg/) databases. Functional‐enrichment analysis, including GO and KEGG, was performed to identify targets significantly enriched in GO terms and metabolic pathways at P‐adjust < 0.05 compared to the whole‐ref genes background. GO functional enrichment and KEGG pathway analysis were carried out using Goatools and Python scipy software, respectively.

### Proliferation Experiment of C2C12 Cells

The proliferation assay of C2C12 cells was conducted using the CCK‐8 cell counting kit (CCK‐8, Dojindo, Japan) to assess the proliferation of cells stimulated by EVs. In brief, 5000 cells were seeded in a 96‐well plate and cultured for 24 h. Subsequently, 10 µL of CCK‐8 solution was added to each well, followed by co‐incubation with the cells for 3 h. Cell proliferation was determined by measuring the absorbance at 450 nm using an enzyme‐linked immunosorbent assay plate reader. The data were expressed as mean ± standard deviation (SD) from three independent replicates.

### Transwell Migration Assay

The migratory behavior of C2C12 cells under the influence of EVs was assessed by using a Transwell assay. In essence, C2C12 cells, suspended in serum‐free medium at a density of 10 × 104 cells per well, were seeded into the upper compartment of the Transwell apparatus (BD Falcon, USA). The cells were permitted to migrate for a duration of 8 h. Subsequent to this period, cells remaining on the upper surface of the membrane were gently wiped away, while those that had traversed to the lower surface were fixed with a 4% solution of paraformaldehyde (PFA). The cells were then stained with a 0.2% solution of crystal violet to facilitate visualization under microscopic examination. The results, reflecting the average number of migrated cells from three independent experiments, are expressed as mean ± standard deviation.

### Immunofluorescence Experiment of C2C12 Cells

The immunofluorescence experiment of C2C12 cells (MYOG, DAPI staining) proceeded as follows: After differentiation in high‐glucose DMEM medium containing 10% FBS for 3 days, cells in logarithmic growth phase were seeded onto coverslips to form a monolayer. Subsequently, cells were cultured in high‐glucose DMEM medium supplemented with 2% horse serum for 7 days. Following this, cells were fixed with 4% paraformaldehyde at room temperature for 30 min, washed three times with 1× PBS for 5 min each. Non‐specific sites were then blocked with 1% bovine serum albumin (BSA) at room temperature for 30 min. Each coverslip was incubated overnight at 4 °C with mouse monoclonal anti‐myogenin primary antibody (1:200 dilution in 1% BSA/PBS) (Beyotime, AF7542, China), followed by three washes with 1× PBS for 5 min each. Subsequently, coverslips were incubated with FITC‐conjugated secondary antibody (anti‐rabbit or anti‐mouse) (1:500 dilution in 1% BSA/PBS) (Beyotime, A0562, China) at room temperature in the dark for more than 1 h, followed by three washes with 1× PBS for 5 min each. Finally, coverslips were stained with DAPI (Beyotime, C1005, China) in the dark for 10 min, mounted with a mounting medium, and observed and photographed under an immunofluorescence microscope.

### Hydrogel's Protective Effect Against ROS in C2C12 Cells

C2C12 cells were seeded in a plate and treated with H_2_O_2_ (400 µmol) for 24 h to establish an oxidative stress microenvironment. Different concentrations of decellularized extracellular matrix materials were then added, and the cells were co‐cultured for 48 h. Cell viability was assessed using the CCK‐8 and Calcein‐AM/PI dual staining method. Intracellular ROS detection was performed using DCFH‐DA as the ROS fluorescent probe. Cells were co‐incubated with different treatments for 30 min, followed by PBS washing. Fluorescence microscopy was used to observe and image the staining of live/dead cells and ROS. Quantitative analysis was conducted using ImageJ software.

### Effect of Hydrogel on Macrophage Polarization

The expression of the macrophage marker CD86 (M1) in RAW cells was detected using flow cytometry. RAW cells were seeded in a 6‐well plate at a density of 1 × 10^6^ cells mL^−1^ with 2 mL per well. After 12 h, the medium was replaced with hydrogel extract, and cells were cultured for an additional 3 days. Subsequently, the cells were scraped off, washed twice with phosphate‐buffered saline (PBS), and thoroughly resuspended in 250 µL of fixation/permeabilization solution, followed by incubation at 4 °C for 20 min. After washing the cells twice with buffer (from the fixation/permeabilization kit, BD), they were incubated with antibody solution containing CD86 (dilution factor 1:100, H2316, Santa Cruz Biotech) at 4 °C for 30 min. After antibody incubation, the cells were washed twice with buffer, resuspended in PBS, and analyzed using a flow cytometer (FACS, AriaII, BD).

### Animal Model Establishment and Grouping

This experiment obtained ethical approval from the Ethics Committee of the Tenth People's Hospital of Shanghai (SHDSYY‐2023‐3825‐3).

The specific steps of the experiment are as follows: A total of 48 male Sprague‐Dawley rats, aged 8 weeks, were utilized for the experiment, 12 rats per group, which were divided into two time points for intervention and sampling: at 6 weeks and 12 weeks post‐surgery. At each time point, 6 rats were taken out for the respective treatments and tissue collection. After sodium pentobarbital anesthesia of SD rats, an incision was made on the left hind limb posterior lateral thigh to expose the sciatic nerve, freeing the sciatic nerve while preserving the branches of the sciatic nerve to the thigh muscles. The proximal end of the nerve was freed to the level of the hamstring tendon, and the distal end was carefully freed to the entry points of the nerve branches into the muscles. The left sciatic nerve was cut off at the distal end below the hamstring tendon, and the proximal end of the nerve was inverted and buried in the nearby hamstring muscle belly. Two nylon sutures were used to secure the outer membrane and fascicles of the distal end of the nerve, which was pulled proximally and fixed to the hamstring tendon, ensuring that the distal end of the sciatic nerve was positioned at the distal end of the hamstring tendon. Before closing the wound, the left hind limb was maximally moved to ensure secure fixation of the traction lines. On the 14th day post‐modeling, the experimental groups were subjected to ultrasound stimulation. After anesthesia, a medical ultrasound therapy station (Careimed, CT2404, China) was used to apply ultrasound stimulation to the tibialis anterior muscle where the hydrogel had been injected (15 minutes, 1 MHz). Subsequently, a second surgery was conducted in the sixth week following the operation to repair the damaged nerves. The rats then underwent continuous rehabilitation training. During the experiment, samples were taken from the anterior tibialis muscle at the sixth and twelfth weeks, respectively. The experimental groups were as follows: Control Group: Rats that underwent surgery without any further intervention. UR‐gel Group: Rats that received an injection of the hydrogel immediately after surgery. UR‐gel + US Group: Rats that received an injection of the hydrogel followed by ultrasound stimulation. EVs@UR‐gel + US Group: Rats that received an injection of the EVs‐loaded hydrogel followed by ultrasound stimulation. Throughout the entire experimental period, the rats were housed in an SPF‐grade experimental animal facility. They were maintained under controlled temperature and humidity conditions, with constant access to water and food. And there were no deaths or exclusions of animals from the experiment.

### In Vivo Fluorescence Assay

A lipophilic fluorescent probe DIR was labeled with EVs, and the prepared material was injected into the anterior tibialis muscle of rats using a 1 mL syringe immediately after establishing the rat nerve injury model. Imaging was performed at 0day, 3day, 7day, 14day, and 21day by using an in vivo imaging system (PerkinElmer). Regions of interest were identified, and the total radiant efficiency of the rats was recorded at a wavelength of 710 nm excitation/760 nm emission.

### Measurement of Sciatic Nerve Function Index (SFI)

A wooden trough with open ends, measuring 60 cm in length, 10 cm in width, and 10 cm in height, was constructed. A piece of white paper weighing 70 g was cut to the same length and width as the trough and laid at the bottom. Rats' hind limbs were colored by dipping them in paint at the ankle joints. The rats were then placed at one end of the trough and allowed to walk toward the other end, leaving 5–6 footprints on each side. The following six parameters were measured for each clear footprint: ETS (injured toe spread), NTS (normal toe spread), EPL (injured print length), NPL (normal print length), EIT (injured intermediary toe spread), NIT (normal intermediary toe spread). These indices were then input into the Bain formula to calculate the SFI. An SFI of 0 indicates normal function, while ‐100 indicates complete damage. The Bain formula is as follows:

(1) $$\mathrm{TSF}\left(\mathrm{toe}\;\mathrm{spread}\;\mathrm{factor}\right)=\left(\mathrm{ETS}-\mathrm{NTS}\right)/\mathrm{NTS}$$

(2) $$\mathrm{PLF}\left(\mathrm{print}\;\mathrm{length}\;\mathrm{factor}\right)=\left(\mathrm{EPL}-\mathrm{NPL}\right)/\mathrm{NPL}$$

(3) $$\mathrm{ITF}\left(\mathrm{intermediary}\;\mathrm{toe}\;\mathrm{spread}\;\mathrm{factor}\right)=\left(\mathrm{EIT}-\mathrm{NIT}\right)/\mathrm{NIT}$$

(4) SFI=109.5TSF−38.3PLF+13.3ITF−8.8

### Measurement of Maximum Isometric Contraction Force

A Kocher needle with a diameter of 1.0 mm was used to secure the distal end of the rat's femur and ankle joint to a wooden board, stabilizing the lower leg segment and maintaining muscle length. Simultaneously, the muscle tendon insertion point of the anterior tibial muscle was surgically exposed. The initial stimulation frequency was set at 10 Hz, with a duration of 0.4 milliseconds, and the voltage was set at 2 V. The force transducer was calibrated using weights of 0 g, 10 g, 20 g, 30 g, and 50 g, respectively. The muscle tendon insertion point of the anterior tibial muscle was connected to the force sensor, and a single stimulation of the proximal end of the sciatic nerve anastomosis was applied using the stimulating electrode, maintaining the aforementioned stimulation parameters. The weight at the muscle tendon insertion point was incrementally increased by 0 g, 5 g, 10 g, 15 g, and 20 g increments (increasing by 5 g each time until the optimal length was determined) to find the maximum force generated by this single stimulation at each weight. The muscle length at this point was considered the optimal length. At the optimal length, the voltage was adjusted to 10 V, and the stimulation frequency was varied at 50 Hz, 100 Hz, 150 Hz, and 200 Hz (starting from 50 Hz and increasing by 50 Hz each time) to measure the maximum isometric contraction force under continuous stimulation of the sciatic nerve at equal lengths.

### Muscle Circumference, Muscle Wet Weight and Histological Examination

The initial length of the muscles at relaxation was measured. Bilateral anterior tibial muscles were excised completely from their origin to insertion points in relaxed muscles. The superficially adherent subcutaneous tissues were carefully removed, then measure the muscle circumference at the thickest part of the muscle and compared it to the unoperated side, and subsequently, the wet weight was measured by using an analytical balance (with a sensitivity of 1 mg, R200D, Germany) and recorded. Frozen sections from the medial heads of the left anterior tibial and gastrocnemius muscles were subjected to Masson's trichrome staining. Acid fuchsin stained muscle fibers red, while aniline blue stained collagen fibers blue.

### Immunofluorescence Staining of Tissue Sections

Tissue sections from the anterior tibial muscle collected at six weeks post‐operation were subjected to immunofluorescence staining. The muscle tissue was fixed in 4% formaldehyde, dehydrated through a sucrose gradient, embedded in OCT compound, and sliced into 12‐micrometer sections. These sections were then blocked at room temperature for 1 h. Subsequently, they were incubated overnight at 4 °C with primary antibodies, including mouse anti‐MyHC (dilution 1:200, Abcam, Cambridge, UK) and rabbit anti‐Col‐1 (dilution 1:200, Abcam, Cambridge, UK). After rinsing with PBS, the sections were incubated with the corresponding secondary antibodies at room temperature in the dark for 2 h. Following another round of PBS washing, the sections were stained with DAPI to visualize the cell nuclei. Finally, fluorescence microscopy was employed for imaging.

### Transcriptome Sequencing and Bioinformatics Analysis

At 6th week post‐operation, total RNA was extracted from denervated and denervated +EVs@UR‐gel anterior tibial muscles using mRNA isolation kit. Subsequently, the RNA integrity was assessed using an Agilent 2100 Bioanalyzer (Agilent Technologies, Santa Clara, CA, USA). Ribosomal RNA was enzymatically digested using the TruSeq Stranded Total RNA and Ribo‐Zero Gold kits. The fragmented RNA was used as a template for cDNA synthesis and library construction with fragment buffer solution. RNA libraries were then subjected to RNA identification using the Agilent 2100 Bioanalyzer. Sequencing was performed using an Illumina sequencer (HiSeqTM 2500 or Illumina HiSeq X Ten). DESeq software was employed for normalizing mRNA counts for each sample and calculating fold changes. Differential expression of reads between the two groups was assessed using a negative binomial distribution test. Finally, genes with fold changes of either >1.5 or ←1.5, with a q‐value < 0.05, were selected as differentially expressed genes. For functional analysis based on Gene Ontology (GO) and Kyoto Encyclopedia of Genes and Genomes (KEGG) pathway analysis, enrichment was considered significant for *p*‐values < 0.05.

## Conflict of Interest

The authors declare no conflict of interest.

## Supporting information



Supporting Information

## Data Availability

The analyzed datasets generated during this study are available from the corresponding author on reasonable request.
