# Supplementary material for: Treatment of Denervated Muscle Atrophy by Injectable Dual‐Responsive Hydrogels Loaded with Extracellular Vesicles
Source: Adv Sci (Weinh). 2025 Jan 21;12(10):2412248. doi: 10.1002/advs.202412248 (PMC11905034; doi:10.1002/advs.202412248)
Supplement: Supplementary file 1 — Supporting Information [file ADVS-12-2412248-s001.docx]

**Supporting Information for**

Treatment of Denervated Muscle Atrophy by Injectable Dual-responsive Hydrogels Loaded with Extracellular Vesicles

Ziheng Bu,^∇^ Jianxing Jing,^∇^ Wei Liu,^∇^ Zhen Fan, Junchao Huang, Zheng Zhou, Jianhai Hu, Jiachang Hong, Daolin Tang, Min Sun,* Jianzhong Du,* and Peng Wu*

**Figure S1.** a) The absorbance of concentrations of standard methylene blue samples. b) Standard fitting curve between MB concentrations and absorbance. c) The temperature variation diagram of ultrasound acoustic thermal effect between SBF and UR gel. d) Photothermal image of SBF and UR gel with an ultrasound for 10 min.

**Figure S2.** a) Light field diagram of HUC-MSCs, scale bar = 100 μm. b) Results of osteogenic, chondrogenic and adipogenic differentiation of HUC-MSCs, scale bar = 100 μm. c) Characteristic surface markers of HUC-MSCs evaluated by flow cytometry.


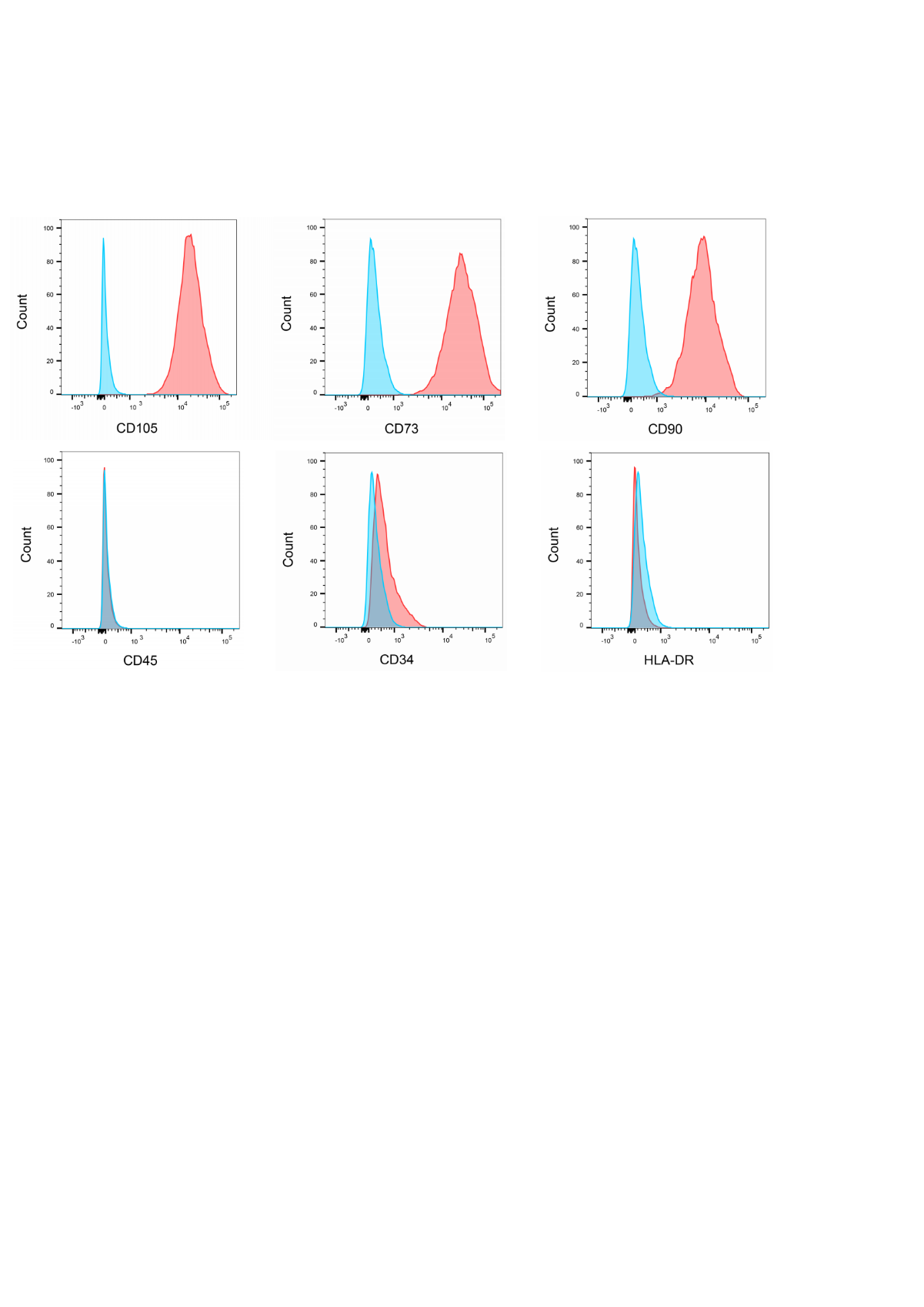


**c**


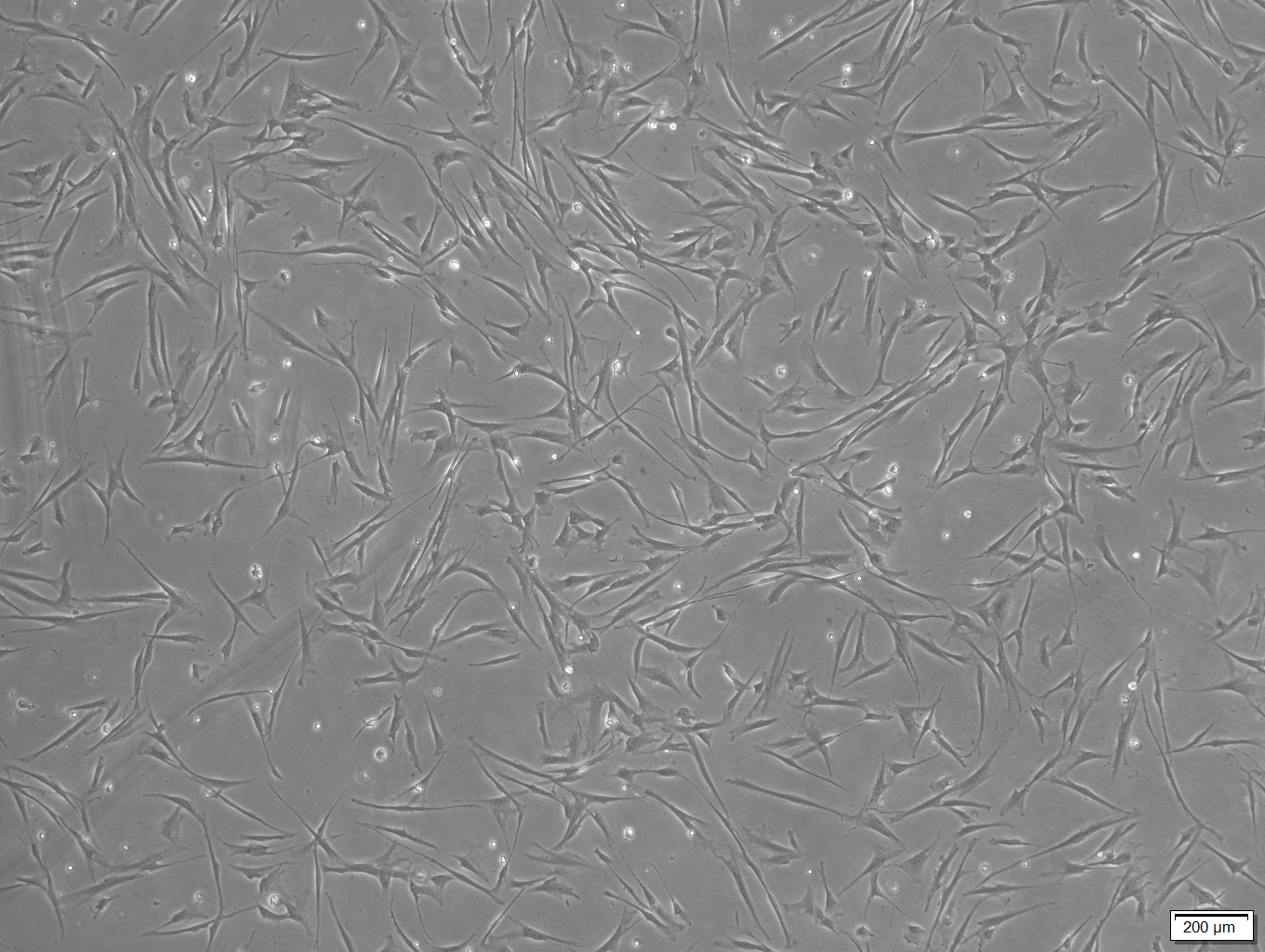


**a**


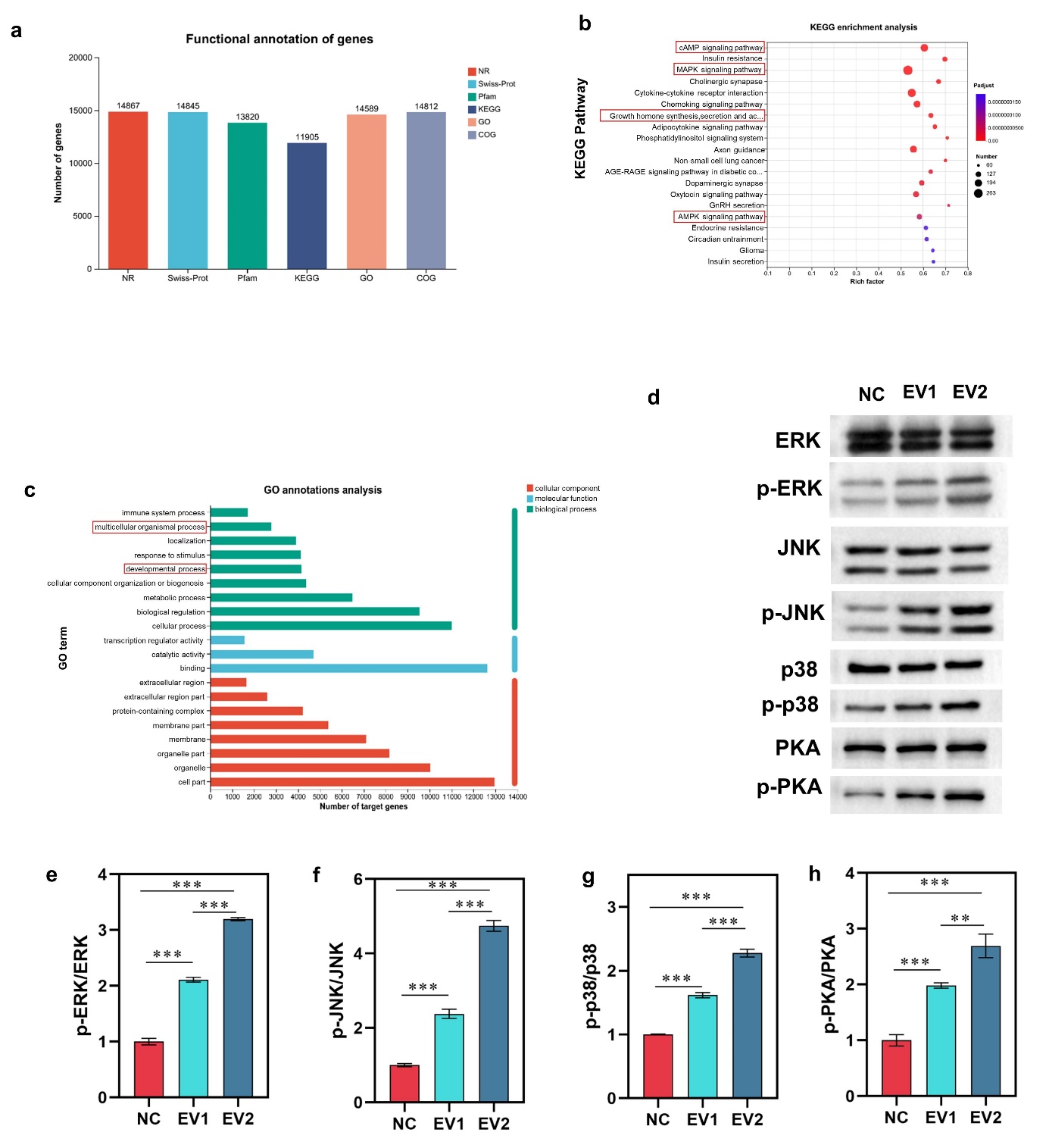


**Figure S3.** a) Analysis of miRNA target gene quantity in commonly used databases. b) Kyoto Encyclopedia of Genes and Genomes (KEGG) target gene analysis shows that target genes are enriched in signaling pathways such as cAMP, MAPK, AMPK. c) Gene Ontology (GO) analysis of target genes. d) Validate the expression and phosphorylation of representative proteins in the MAPK, cAMP, and AMPK signaling pathways through WB. e-h) Quantitative analysis of related proteins and their phosphorylation status.


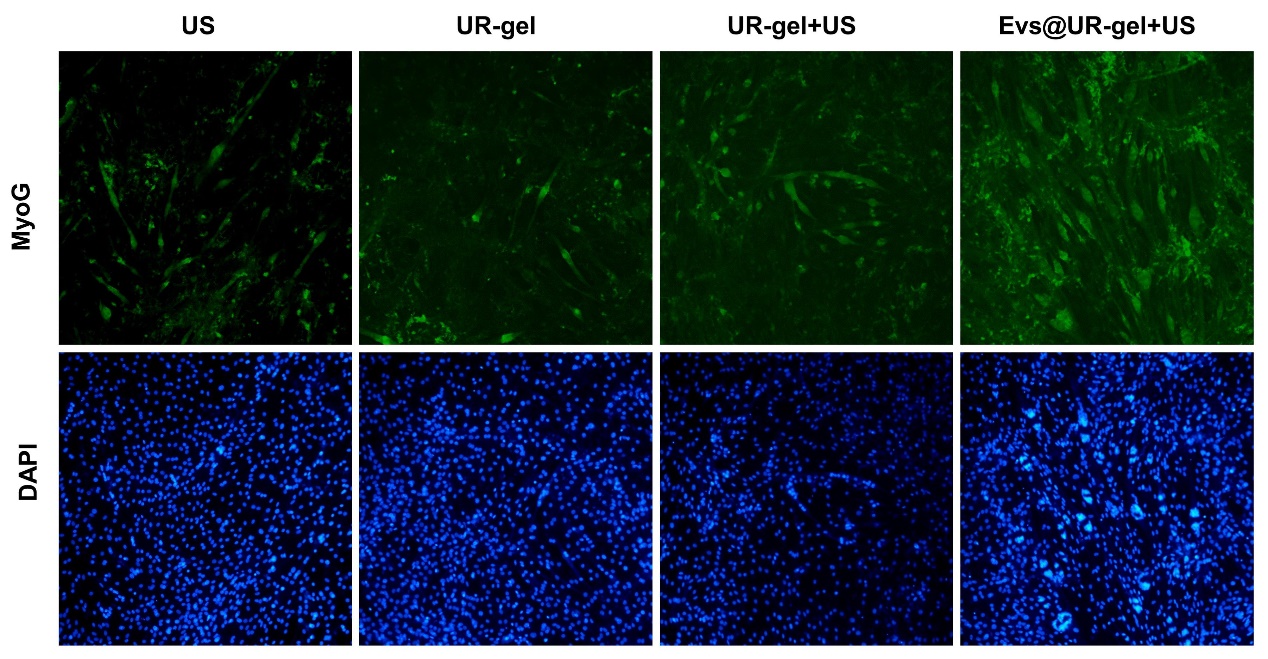


**Figure S4.** C2C12 myogenic differentiation MyoG and DAPI immunofluorescence staining, scale bar = 100 μm.

**Figure S5.** a) Protective effect of UR-gel against H_2_O_2_-induced cell death in C2C12, scale bar = 100μm. b) RAW 264.7 cells immunofluorescent staining of CD86 and DAPI, scale bar = 100 µm.

**Figure S6.** a) Fluorescence plot of EVs distribution in vivo over time, b) Quantitative analysis of Remaining EVs.


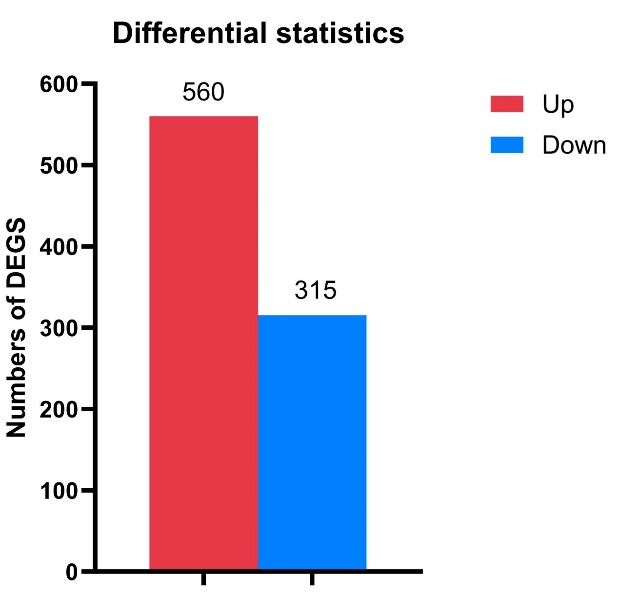


**Figure S7.** Comparison of differentially expressed genes (DEGs) in tibialis anterior muscle tissue between control and EVs@UR-gel+US groups post-modeling week 6.
